# The Cyanobactin Heterocyclase Enzyme: A Processive Adenylase That Operates with a Defined Order of Reaction**

**DOI:** 10.1002/anie.201306302

**Published:** 2013-11-08

**Authors:** Jesko Koehnke, Andrew F Bent, David Zollman, Kieran Smith, Wael E Houssen, Xiaofeng Zhu, Greg Mann, Tomas Lebl, Richard Scharff, Sally Shirran, Catherine H Botting, Marcel Jaspars, Ulrich Schwarz-Linek, James H Naismith

**Affiliations:** Biomedical Science Research Complex, University of St AndrewsNorth Haugh, St Andrews, KY16 9ST (UK); Marine Biodiscovery Centre, Department of Chemistry, University of AberdeenMeston Walk, Aberdeen, AB24 3UE (UK); Institute of Medical Sciences, University of AberdeenAberdeen AB25 2ZD (UK)

**Keywords:** adenylation, cyanobactins, heterocyclase, ribosomal peptide pathways

Heterocyclic rings are a recurring and iconic motif in organic chemistry. The introduction of five-membered rings into protein backbones is synthetically challenging, but many peptide-based biologically active natural products contain such Ser-, Thr-, and Cys-derived heterocycles[1] and therefore a facile route to their introduction is highly desirable. More generally thiazolines and oxazolines, as well as their oxidized forms (thiazoles and oxazoles), are found in a variety of approved drugs, drug leads, and toxins.[2]

**Scheme 1:**
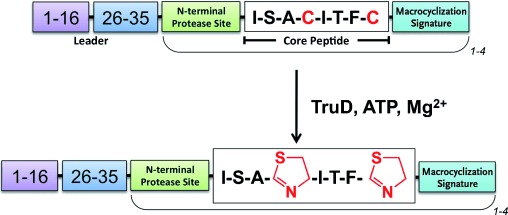
Heterocyclization reaction catalyzed in the cyanobactins. TruD efficiently processes cysteines and selenocysteine.[8] TruD operates on core peptides of seven or eight residues; a single precursor peptide can contain up to four core peptides each with different sequences. The immediate sequence context and position (relative to the leader) of the target cysteine is variable.

The cyanobacterium *Prochloron spp.* produces multiple macrocyclic cyanobactins, which are known as the trunkamides (seven or eight residues) and the patellamides (eight residues). These natural products originate from two different ribosomal precursor peptides (denoted TruE and PatE), and each ribosomal peptide contains multiple distinct core peptides. In TruE (and PatE), each of the core peptides is flanked at the N-terminus by a conserved five-residue protease signature[3] and at the C-terminus by a conserved macrocyclization signature (A/SYDG; Scheme 1). It is the core peptides that go on to become the different natural products. In trunkamides and patellamides (as well as other cyanobactins) these products possess multiple heterocyclic cysteine, and/or serine and threonine amino acids. All cyanobactin ribosomal precursor peptides possess (along with the core peptides and their flanking regions) a conserved thirty-to forty-residue N-terminus (Scheme 1). This N-terminal leader, which is discarded during processing is thought to be essential for heterocyclization.[4] Heterocyclization of the multiple cysteine residues in TruE is carried out by the single enzyme, TruD, which by definition is processive. The amino acid sequence of the core peptide is variable (Supporting Information, Figure S1) and thus TruD must be in part insensitive to the immediate sequence context of the target cysteine (Scheme 1). Further, the positions of the target cysteines in TruE relative to the leader vary, both within and between core peptides. This flexibility suggests that TruD could be valuable for synthetic chemists. The corresponding enzyme in the patellamide pathway is denoted PatD. TruD and PatD process cysteine residues[4, 5] in the other enzymes’ precursor peptide substrate equally well; TruD processes serine and threonine residues less well than PatD.[4, 5] The interchangeability of enzymes mirrors the high degree of sequence identity in the enzymes (Figure S2) and substrate N-terminal leader (Figure S1).

The heterocyclization of cysteine to form thiazoline as part of the microcins (thiazole/oxazole-modified microcins, or TOMMs) has recently been studied and the responsible ATP Mg^2+^ dependent heterocyclase BalhD identified.[6] BalhD is homologous to the C-terminal 400 residues of TruD, whilst a second protein BalhC matches the N-terminal 300 residues (Figure S2). When both BalhD and BalhC are present heterocyclization of the microcin substrate is accelerated and only when both proteins are present can phosphate release be robustly measured. Pyrophosphate production by BalhD was ruled out and BalhD was proposed to operate by a kinase type mechanism in which a hemiorthoamide intermediate is phosphorylated[6, 7] (Figure S3).

The cyanobactin class of heterocyclases, exemplified by TruD, possess an almost unique combination of processivity, specificity, chemical versatility, and promiscuity, which thus makes them a fascinating subject for study. We show by biochemical assay that TruD is an adenylase, not a kinase, and that TruD processes cysteines in a defined order, which we describe. We demonstrate that entire substrate leader can be removed and TruD will process a single specific cysteine residue. However, we establish the role of leader is to permit processivity through a balance of recognition.

**Figure 1:**
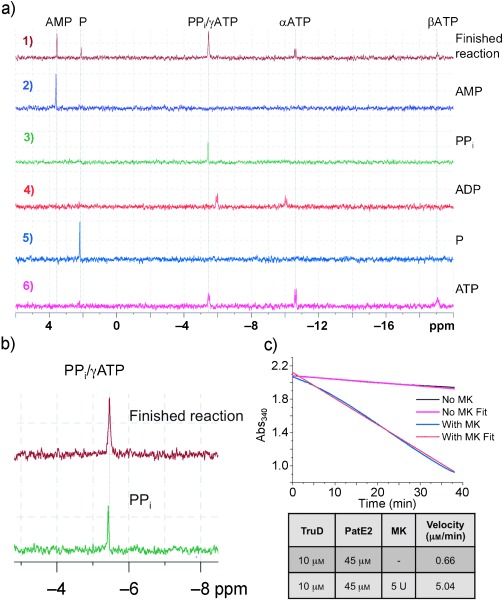
PatE2 TruD reaction. a) ^31^P NMR of a heterocyclization reaction, authentic AMP, pyrophosphate (PP_i_), ADP, phosphate (P), and ATP. AMP and PP_i_ predominate at end of reaction with small amounts of phosphate and unreacted ATP present; no ADP is observed. b) Expansion of the PP_i_ peak in the final reaction and PP_i_ standard. c) Decrease of absorbance in the coupled NADH assay in the presence (blue) and absence (black) of myokinase (MK). Reaction rates were calculated from fitted curves (red and magenta, respectively). Addition of MK accelerates the apparent rate by a factor of about 8.

TruD overexpressed in *E.* *coli* BL21 (DE3) cells and purified to homogeneity (as judged by SDS-PAGE) using established procedures[8] was used for all analyses. TruD (5 μM), when incubated with Mg^2+^, ATP (5 mM) and an engineered PatE2 (single core peptide sequence ITACITFC, Figure S1; 100 μM) results in a loss of mass in PatE2 of two water molecules (Figure S4a). Mass spectrometry (MS) fragmentation data confirms the formation of heterocycles,[5, 8] validating TruD activity and substrate. 2.2 molar excess of ATP (1.1 for cysteines) was added to PatE2 at 20 °C and incubated with TruD for 8 h and immediately the product of the reaction was analyzed by 1D ^31^P NMR spectroscopy. Authentic AMP, ADP, ATP, pyrophosphate, and phosphate in the same buffer at the same concentration and pH were used as standards (Figure 1 a,b). Two new peaks matching AMP and pyrophosphate were identified in the product mixture. Unreacted ATP and a smaller amount of phosphate were observed, but no ADP. We repeated the experiment at 10 °C and recorded spectra every 30 min for the first 12 h and every 2 h thereafter (Figure S5a). This time-course confirms AMP and pyrophosphate production (and small amount of phosphate). A control incubation of the mixture with ADP reveals that it only slowly degrades to AMP. These data can only be explained by an adenylation mechanism and rules out a kinase mechanism. Repeating the NMR after overnight incubation or at a temperature >25 °C shows only phosphate (Figure S5b), a fact we attribute to pyrophosphate degradation rather than a time-or temperature-dependent change in mechanism.

ATP, ADP, and AMP bind to TruD with *K*_D_ values of 19, 20, and 12 μM, respectively (Figure S5c), but as ADP decomposes slowly in a dissipated exothermic manner, the corresponding *K*_D_ value is an apparent value obtained by analyzing the rapidly emerging endothermic parts of the ITC signals. Although dTTP does not bind in ITC experiments, we find it and GTP can catalyze heterocyclization of cysteine (Figure S5 d–f); BalhD has also been shown to use GTP.[6]

The coupled NADH assay reported in the study of the adenylase enzyme AcsD[10] uses lactate dehydrogenase to oxidize NADH during the reduction of pyruvate. Pyruvate is generated by pyruvate kinase, which requires ADP. The assay has a background rate in the absence of any substrate,[10] which for a slow enzyme such as TruD is significant. The addition of TruD and/or PatE2 to the mixture did not increase the rate of NADH consumption above background, indicating no enzymatic production of ADP. Addition of myokinase, which converts AMP and ATP into ADP, accelerates the rate of NADH consumption to 5.04 μM min^−1^ (Figure 1 c), confirming enzymatic production of AMP consistent with an adenylase mechanism.

**Figure 2:**
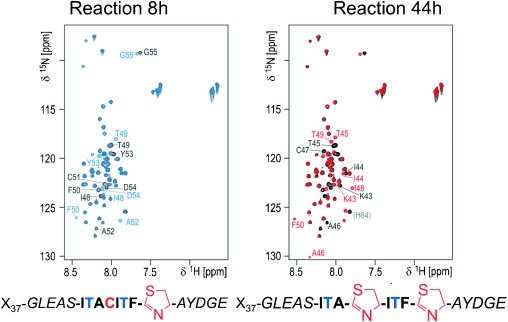
PatE2-TruD reaction monitoring and binding analysis. Overlay of HSQC spectra of ^15^N-PatE2 in presence of ATP/Mg^2+^ without TruD (black cross-peaks) and 8 h (left panel, blue cross-peaks) or 44 h (right panel, red cross-peaks) after addition of the enzyme. Resonances that disappear in the course of the transformation are labeled black; emerging resonances are labeled blue (8 h) and red (44 h). The cross-peak for C-terminal His64 undergoes a gradual chemical shift change in the course of the reaction (fast chemical exchange rather than the slow exchange observed for other affected resonances).

Time-course MS experiments showed that although both cysteines were heterocyclized after about 60 min at 37 °C, there appeared to be an accumulation of a mono-heterocyclized intermediate during the reaction (Figure S4b). Using standard triple-resonance experiments, we assigned the spectra (except four of the six histidine residues of the C-terminal tag) of uniformly ^13^C, ^15^N-labeled PatE2 (Figure S6). The sharp, poorly dispersed NMR signals and ^13^C chemical shifts indicate PatE adopts a natively unfolded state in aqueous buffer (compare with the helical arrangement of N-terminal leader peptide in organic solvent[11]). Real-time ^1^H-^15^N HSQC-NMR reaction monitoring using uniformly ^15^N-labeled PatE2 showed a specific order with the first heterocyclization event characterized by the disappearance of eight cross-peaks (assigned to residues 48–55) and the emergence of seven new cross-peaks (Figure 2). In the second heterocyclization event, eight cross-peaks (residues 43–50) disappeared (three of which appeared with first heterocyclization) and seven new peaks appeared (Figure 2).

Heterocyclization of cysteine induces large changes in ^1^H^N^, ^15^N^H^, C^α^, and C^β^ chemical shifts (Figure S6). This allowed us to establish that the terminal cysteine, C51, heterocyclizes first, followed by the slower heterocyclization of the internal cysteine C47 (Figure 2; Supporting Information, Figure S6). The reaction was highly temperature-dependent with completion in under 5 h at 20 °C, but still incomplete after 44 h at 10 °C (Figure 2). The internal cysteine of the PatE2 mutant C51P (chosen to mimic the mono-heterocyclized intermediate) was processed (within error) at the same rate as the internal cysteine in PatE2 (Figure S7). However, the internal cysteine of the C51A PatE2 mutant is processed very slowly (Figure S7). The conformational change at the C-terminus of core peptide introduced upon formation of the five-membered ring is thus critical for TruD to be able to process the internal cysteine of the core peptide.

We incubated a uniformly ^15^N-labeled PatE with a core peptide with three cysteines (sequence ICACITFC (PatE3C)) and monitored the reaction by HSQC NMR at 10 °C and observed that C51 reacts first, followed by C47 and finally C45 (Figure S8). TruD thus works from the C-terminus within the core, but the accumulation of the intermediates shows that they are released and rebound, rather than held on the enzyme. The first two heterocyclizations of PatE3 occur on an identical timescale (at both 10 °C and 37 °C) to the two-cysteine PatE2 substrate, but the third heterocyclization is slower than the second heterocyclization (which is slower than the first).

**Figure 3:**
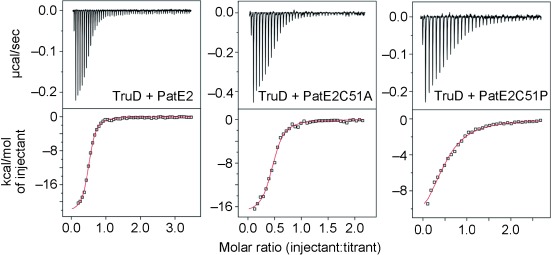
ITC data obtained for the injection of PatE and PatE variants into TruD solutions. The top panels show raw data representing the response to injections, the bottom panels show integrated heats of injections (□) and the best fit (—) to the one-site model (origin).

PatE2 binds very tightly to TruD (*K*_D_ 80 nM) (Figure 3), but titrating TruD into ^15^N-PatE2 shows that ^1^H, ^15^N-HSQC cross-peaks of residues 1–15 are unaffected and thus do not bind to TruD (Figure S6e); they might therefore have no role in substrate recognition.

Using MS fragmentation analysis and ITC (where the substrate was sufficiently soluble), we sought to define the role of precursor peptide residues outside the core peptide in controlling substrate recognition. A synthetic peptide with the first 25 residues of PatE2 deleted (Δ25PatE) retains the most conserved leader region, residues 26-35 (denoted “minimal leader”), and was processed by TruD only slightly more slowly than PatE2 (judged by MS; Figure S9). Insertion of up to six amino acids between the N-terminal protease signature, and the core peptide permitted normal heterocyclization (Figure S7, Table S1). Four further synthetic peptides were tested: Δ37PatE (retains five-residue protease signature) Δ42PatE (no leader), the eight-residue core peptide itself and the eight-residue core peptide with an additional C-terminal Gly (Figure S9). No reaction is seen with core peptide alone or core peptide with C-terminal Gly. The terminal cysteine only is however processed in both Δ37PatE and Δ42PatE peptides (more slowly than Δ25PatE but within an order of magnitude). From these data we conclude that recognition of the C-terminal cysteine of the cassette does not require the leader.

Radical mutants of individual residues within the center of the minimal leader region revealed S30F had no effect but mutants L29R and E31R did not allow processing of the PatE2 to completion. In both cases, we observed the mono-heterocyclized intermediate along with the product but no unreacted substrate, indicating that the rate of the second heterocyclization is significantly slower in these mutants but the rate of the first heterocyclization is largely unaffected. Both mutations significantly decreased binding affinity (Figure S7). Mutations G38I, L39N, A41I, and S42C within GLEAS protease signature had little or no effect on processing (first and second heterocyclization; E40R was insoluble in our hands); although the binding was decreased (Figure S7). The “new” cysteine of S42C was not processed by TruD. Y53A and D54R (in the C-terminal macrocyclization signature) were processed normally. Processivity (heterocyclization of the second or internal cysteine of the core peptide), although very sensitive to changes in the minimal leader, is insensitive to changes in the core peptide flanking regions. We explored more radical mutants S42Q and A52D, which immediately flank the core peptide; both mutants significantly disrupted binding and processing. In contrast to changes in the leader peptide however, these mutations primarily produced either fully heterocyclized product or unreacted substrate. This suggests that these mutations affect the rate of the first heterocyclization (C-terminal cysteine), which has now become rate-limiting, but the mutations do not significantly perturb heterocyclization of the internal cysteine (the processivity). The PatE2 A52P mutant has the internal cysteine processed first and only very slowly has the terminal cysteine processed. ITC shows that PatE2 C51A binds almost as tightly as PatE2, but as the internal cysteine is processed extremely slowly, we conclude this binding is non-productive; the Ala at position 51 is bound at the active site. The PatE2 C51P mutant, which mimics the mono-heterocyclized intermediate and permits normal processing of the internal cysteine, binds much more weakly (1500 nM). We suggest this weaker binding allows the internal cysteine to access the active site.

**Figure 4:**
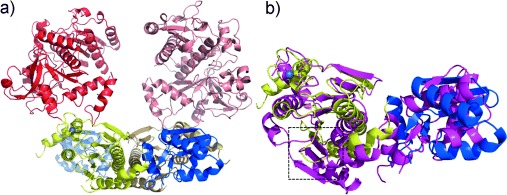
Structure of TruD. a) Structure of the biological TruD dimer. Domain 1 (blue), domain 2 (yellow), domain 3 (red), and the covalently bound Zn (gray sphere in domain 2, near domain 3) are shown. The dimer partner is represented in corresponding faded colors. b) Structural alignment of TruD (domain 1 blue, domain 2 yellow) with MccB (magenta, PDB 3H5A). The MccB ATP binding site is boxed.

The structure of TruD was determined to 2.9 Å (PDB 4BS9) and can be decomposed into three domains (domain 1 residues 2–85, domain 2 residues 86–321, domain 3 residues 323–781), which combine to form an extended, curved molecule that exists as a dimer (Figure 4 a).

Domains 1 and 2 of TruD share 21 % sequence identity and structural fold with the enzyme MccB, an adenylase found in the microcin C7 antibiotic production pathway (MccB catalyzes two successive adenylation reactions; Figure S10).[12] Pairwise superposition of TruD with the MccB structure gives a Cα rmsd of 5.4 Å over 219 structurally equivalent residues (MccB lacks the third domain; Figure 4 b; Supporting Information, Figure S11); individual domain superpositions are much lower due to a domain rotation (2.6 Å over 80 residues and 2.0 Å over 152 residues). TruD and MccB possess a similar dimeric arrangement (Figure 4 a) and both share two CXXC motifs in domain 2 that bind a Zn^2+^ ligated in a tetrahedral arrangement of sulfur atoms. Both motifs are conserved in cyanobactin heterocyclases and these motifs (and associated zinc) are a conserved feature of one class of adenylating enzymes. The coordinatively saturated zinc plays a structural role only12b, [13] in this class of adenylating enzymes.

Domain 2 of TruD shares the fold of this class of adenylating enzymes; other members include human ubiquitin-activating enzyme 5 (1.9 Å rmsd over 134 Cαs) and thiamine pyrophosphate biosynthetic enzyme ThiF (2.1 Å over 142 Cαs). There is, however, a subtle topology difference; all the adenylating enzymes that share a fold with TruD have an insertion between the first and second strand of the central β-sheet relative to TruD domain 2. Significantly this means that the ATP binding site of MccB is not fully conserved in TruD (Figure S11). The ATP-bound structure of MccB (PDB 3H5N) would suggest R123, K190, R221, and R253 of TruD as possible ATP binding residues. R123A retained native activity and the three other TruD mutants were insoluble. D165 in TruD is conserved both in heterocyclases and in the adenylase superfamily but the TruD mutant D165A retained native activity. Structural data of course do not establish the mechanism but they are entirely consistent with TruD being an adenylase enzyme. The 20 % sequence identity of BalhC to TruD (including the two CXXC motifs; Figure S2), establishes that BalhC will have the same fold as domains 1 and 2 of TruD and thus its fold will belong to the same class of adenylating enzymes.

Domain 3 of TruD is a novel fold and has a large negatively charged central cleft (Figure S11). In our hands the third domain of TruD or PatD, expressed separately, is insoluble, but by proteolysis of PatD (highly homologous to TruD) we were able to purify part of the third domain (residues 410–784). In contrast to full-length PatD, which is active, the fragment possessed no catalytic or substrate binding activity (Figure S12). BalhD[6] with 23 % identity will have the same fold as TruD domain 3 and thus possesses an entirely new fold for kinase chemistry.

**Scheme 2:**
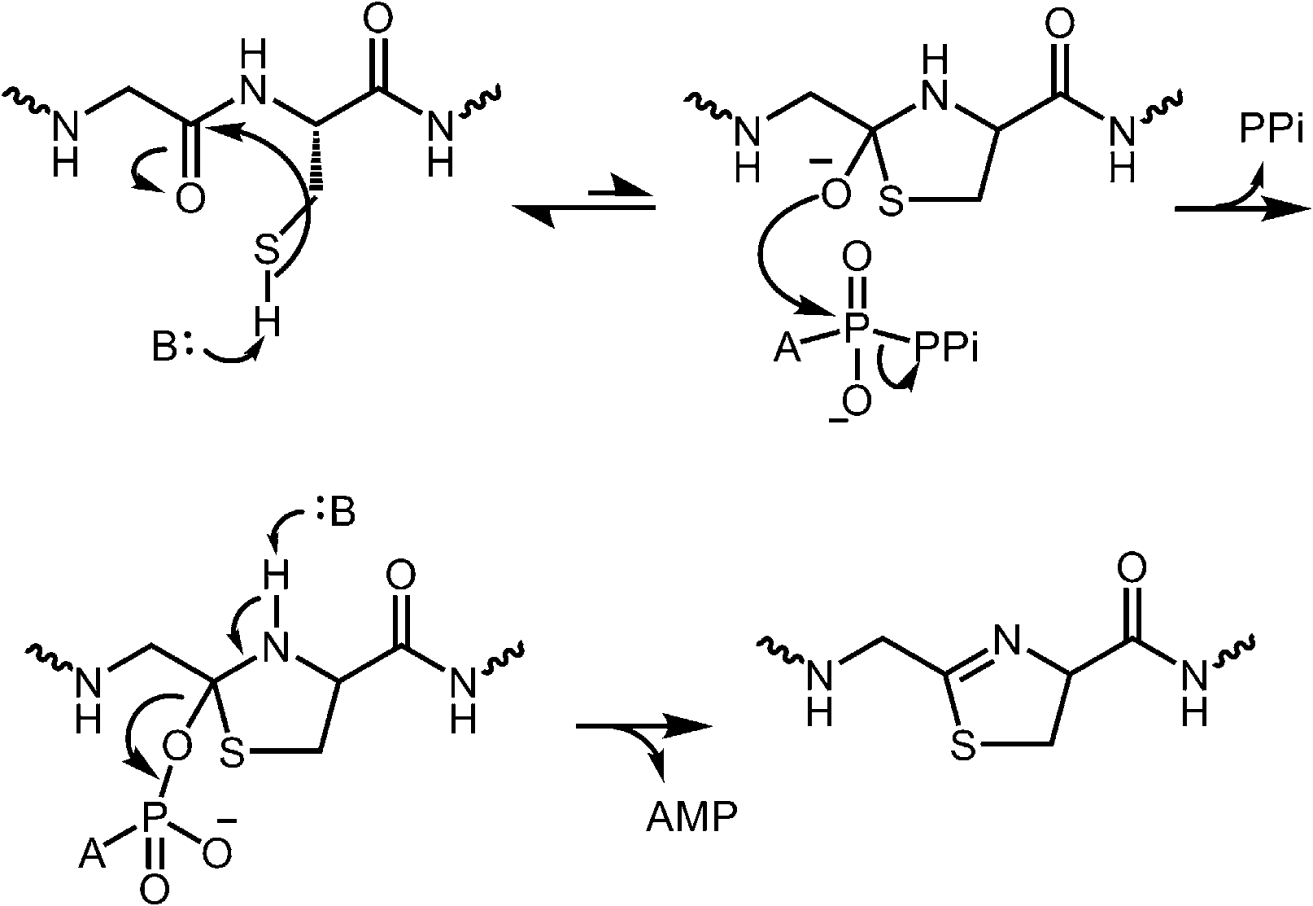
Proposed mechanism for heterocyclization by the TruD class of enzyme. Abstraction of the sulfhydryl proton leads to the formation of a hemiorthoamide, which is adenylated. Irreversible elimination of AMP leads to the formation of the thiazoline. PPi=pyrophosphate.

Biochemical analysis of TruD conclusively rules out a kinase mechanism for this enzyme as there is no ADP production. As both AMP and pyrophosphate are directly detected during turnover, the data can only be interpreted as an adenylation-type mechanism. We did observe phosphate (rather than pyrophosphate) at higher temperature or after prolonged incubation, but this is consistent with degradation of the reactive pyrophosphate. Chemical reasoning would suggest the carbonyl oxygen of amides is too unreactive a nucleophile to react directly with ATP. In any event, such a mechanism would seem to invite futile ATP consumption (adenylation of carbonyl followed by hydrolysis), which is not observed. The most plausible mechanism therefore requires the formation of a five-membered hemiorthoamide ring in a reversible step (Scheme 2), proposed by Dunbar et al.[6] in the BalhD mechanism. The general base required for the first step could come from PatE but the observations that the entire leader region can be removed, and the conserved flanking residues extensively mutated argues against this. We suggest the hemiorthoamide intermediate attacks the α-phosphate of ATP displacing pyrophosphate giving an adenylated hemiorthoamide. This highly reactive intermediate decomposes to thiazoline and by rapid irreversible elimination of AMP (Scheme 2).

The difference in mechanism of phosphorylation versus adenylation between BalhD and TruD is unexpected; especially given their sequence and by extension structural homology and their shared chemical reaction. We speculate that BalhD and the third domain of TruD both catalyze the reversible formation of the hemiorthoamide ring. In TruD, domain 2 then adenylates the intermediate whilst in the TOMM pathway, BalhC docks to BalhD and somehow accelerates the phosphorylation. These data suggest that heterocyclases join a very small number of enzymes exemplified by the type I and type II aldolases, which catalyze the same reaction, share a fold yet operate by entirely different chemical mechanisms.

**Scheme 3:**
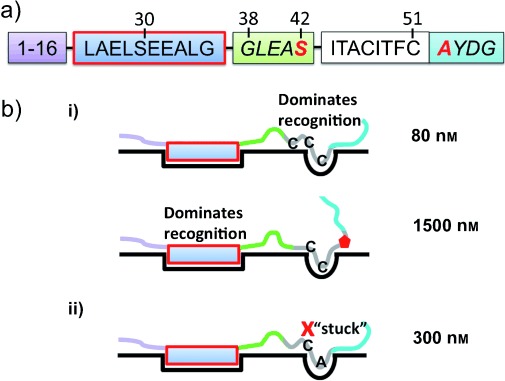
Key residues for PatE/TruD interaction and required order of heterocyclization. a) The precursor peptide containing a single core peptide. Residues essential for the formation of internal heterocycles (processivity) are in the red box, residues whose mutation interferes with the formation of the first heterocycle are highlighted in red. Some residue numbers are given. b) The order of heterocyclization reaction. i) Unmodified substrate binds tightly to the enzyme permitting the first heterocyclization; recognition of substrate does not rely on leader. Formation of the first heterocycle greatly reduces binding affinity allowing successive heterocyclization reactions within the core peptide from the C-terminus; recognition now relies on leader (red box). ii) Mutation of the terminal core peptide cysteine to alanine (C51A) leads to tight binding in a “stuck” conformation unsuitable for processive heterocyclization.

TruD and its homologues have to process multiple (>8) cysteines scattered throughout a larger precursor peptide. NMR showed TruD operates in strict order with the terminal cysteine in the core peptide reacting first; the next most C-terminal cysteine then reacts, and so on (Scheme 3), thus avoiding a combinatorial array of intermediates. The starting material binds tightly (80 nM) whilst the mono-heterocyclized intermediate (mimicked by PatE2 C51P) binds more weakly (*K*_D_ 1500 nM). Thus the intermediate only binds once all the initial substrate is processed. The requirement for a five-membered ring at the end of the core peptide for processing mirrors the situation for the macrocyclase PatG,[14] but for the opposite reason. In the macrocyclase the ring is essential for binding of the substrate, but for the heterocyclase it is essential to prevent unproductive substrate binding at an already processed residue: a form of chemical check point (Scheme 3).

The entire leader peptide could be dispensed with and the terminal cysteine still processed (something similar has been reported in the TOMM pathway[7]). This suggests somewhat special recognition for the terminal cysteine focused on its immediate environment, consistent with our mutational analysis (Scheme 3). What is the role of the leader sequence? We have shown that the leader sequence (crucially residues 26–37) is required for processivity (heterocyclization of subsequent cysteines). We advance a model in which substrate recognition involves both the target residue and the leader, the contribution of each of these to overall substrate binding shifts during processing and it is this differential binding that controls the order of heterocyclization.

As multiple core peptides share a single leader, the signatures flanking the core peptide can be mutated extensively and insertion of residues between the core and leader can be tolerated; we conclude that the spatial relationship between the minimal leader and target residue is highly flexible. This separation of recognition and catalysis is rare and should help drive the use of the enzyme in chemical synthesis.
