# Supplementary material for: The Cyanobactin Heterocyclase Enzyme: A Processive Adenylase That Operates with a Defined Order of Reaction
Source: Angew Chem Int Ed Engl. 2013 Nov 8;52(52):13991–6. doi: 10.1002/anie.201306302 (PMC3995012; doi:10.1002/anie.201306302)
Supplement: Supplementary file 1 [file anie0052-13991-sd1.pdf]

Angewandte Chemie
Eine Zeitschrift der Gesellschaft Deutscher Chemiker

Supporting Information

© Wiley-VCH 2013

69451 Weinheim, Germany

The Cyanobactin Heterocyclase Enzyme: A Processive Adenylase That Operates with a Defined Order of Reaction**

*Jesko Koehnke, Andrew F. Bent, David Zollman, Kieran Smith, Wael E. Houssen, Xiaofeng Zhu, Greg Mann, Tomas Lebl, Richard Scharff, Sally Shirran, Catherine H. Botting, Marcel Jaspars, Ulrich Schwarz-Linek, and James H. Naismith**

anie_201306302_sm_miscellaneous_information.pdf

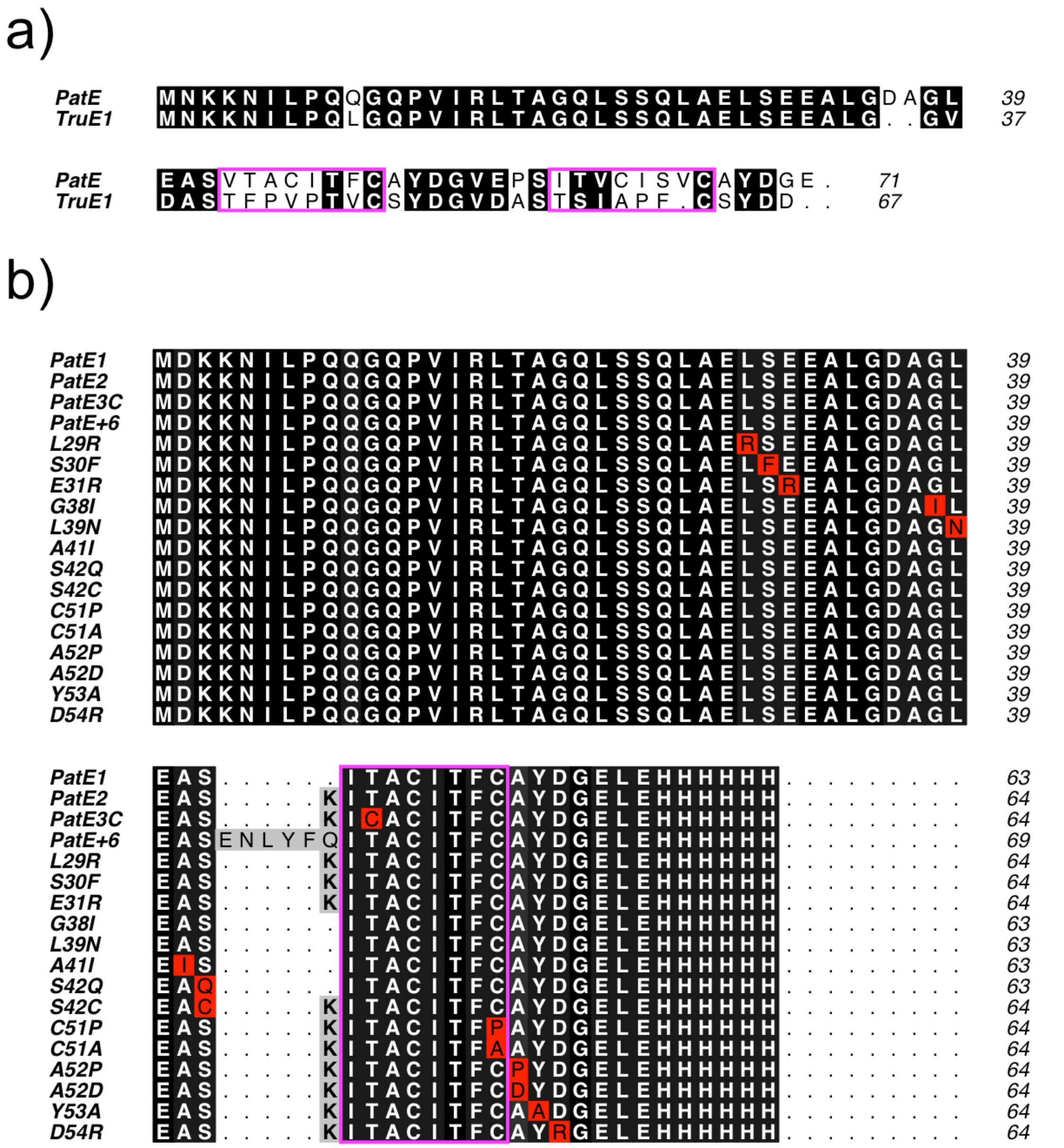

Supplementary Figure 1: a) Sequence alignment of PatE with TruE. Conserved residues are highlighted in black, the core peptides are boxed in magenta. b) Alignment of all PatEs used in this study. Point mutations are highlighted in red, insertions in grey, the core peptides are boxed in magenta.

```
TruD  MQPTALQIKPHFHVEIIEPKQVYLLGEQGNHALTGQLYCQILPFLNG   47
PatD  MQPTALQIKPHFHVEIIEPKQVYLLGEQGNHALTGQLYCQILPFLNG   47

TruD  EYTREQIVEKLDGQVPEEYIDFVLSRLVEKGYLTEVAPELSLEVAAF   94
PatD  EYTREQIVEKLDGQVPEEYIDFVLSRLVEKGYLTEVAPELSLEVAAF   94

TruD  WSELGIAPSVVAEGLKQPVTVTTAGKGIREGIVANLAAALEEAGIQV  141
PatD  WSELGIAPSVVAEGLKQPVTVTTAGKGIREGIVANLAAALEEAGIQV  141

TruD  SDPRDPKAPKAGDSTAQLQVVLTDDYLQPELAAINKEALERQQPWLL  188
PatD  SDPKAPKAPKAGDSTAQLQVVLTDDYLQPELAAINKEALERQQPWLL  188

TruD  VKPVGSILWLGPLFVPGETGCWHCLAQRLQGNREVEASVLQQKRALQ  235
PatD  VKPVGSILWLGPLFVPGETGCWHCLAQRLRGNREVEASVLQQKRALQ  235

TruD  ERNGQNKNGAVSCLPTARATLPSTLQTGLQWAATEIAKWMVKRHLNA  282
PatD  ERNGQNKNGAVSCLPTARATLPSTLQTGLQWAATEIAKWMVKRHLNA  282

TruD  IAPGTARFPTLAGKIFTFNQTTLELKAHPLSRRPQCPTCGDRETLQR  329
PatD  IAPGTARFPTLAGKIFTFNQTTLELKAHPLSRRPQCPTCGDQEILQR  329

TruD  RGFEPLKLESRPKHFTSDGGHRAMTPEQTVQKYQHLIGPITGVVTEL  376
PatD  RGFEPLKLESRPKHFTSDGGHRATTPEQTVQKYQHLIGPITGVVTEL  376

TruD  VRISDPANPLVHTYRAGHSFG.SATSLRGLRNVLRHKSSGKGKTDSQ  422
PatD  VRISDPANPLVHTYRAGHSFGSSAGSLRGLRNTLRYKSSGKGKTDSQ  423

TruD  SRASGLCEAIERYSGIFQGDEPRKRATLAELGDLAIHPEQCLHFSDR  469
PatD  SRASGLCEAIERYSGIFLGDEPRKRATLAELGDLAIHPEQCLHFSDR  470

TruD  QYDNRESSNER.ATVTHDWIPQRFDASKAHDWTPVWSLTEQTHKYLP  515
PatD  QYDNRDALNAEGSAAAYRWIPHRFAASQAIDWTPLWSLTEQKHKYVP  517

TruD  TALCYYRYPFPPEHRFCRSDSNGNAAGNTLEEAILQGFMELVERDSV  562
PatD  TAICYYNYLLPPADRFCKADSNGNAAGNSLEEAILQGFMELVERDSV  564

TruD  CLWWYNRVSRPAVDLSSFDEPYFLQLQQFYQTQNRDLWVLDLTADLG  609
PatD  ALWWYNRLRRPEVELSSFEEPYFLQLQQFYRSQNRELWVLDLTADLG  611

TruD  IPAFVGVSNRKAGSSERIILGFGAHLDPTVAILRALTEVNQIGLELD  656
PatD  IPAFAGLSRRTVGSSERVSIGFGAHLDPKIAILRALTEVSQVGLELD  658

TruD  KVSDESLKNDATDWLVNATLAASPYLVADASQPLKTAKDYPRRWSDD  703
PatD  KVPDEKLDGESKDWMLEVTLETHPCLAPDPSQPRKTANDYPKRWSDD  705

TruD  IYTDVMTCVEIAKQAGLETLVLDQTRPDIGLNVVKVIVPGMR.FWSR  749
PatD  IYTDVMACVEMAKVAGLETLVLDQTRPDIGLNVVKVMIPGMRTFWSR  752

TruD  FGSGRLYDVPVKLGWREQPLAEAQMNPTPMPF  781
PatD  YGPGRLYDVPVQLGWLKEPLAEAEMNPTNIPF  784
```

Supplementary Figure 2A: Sequence alignment of TruD with PatD.

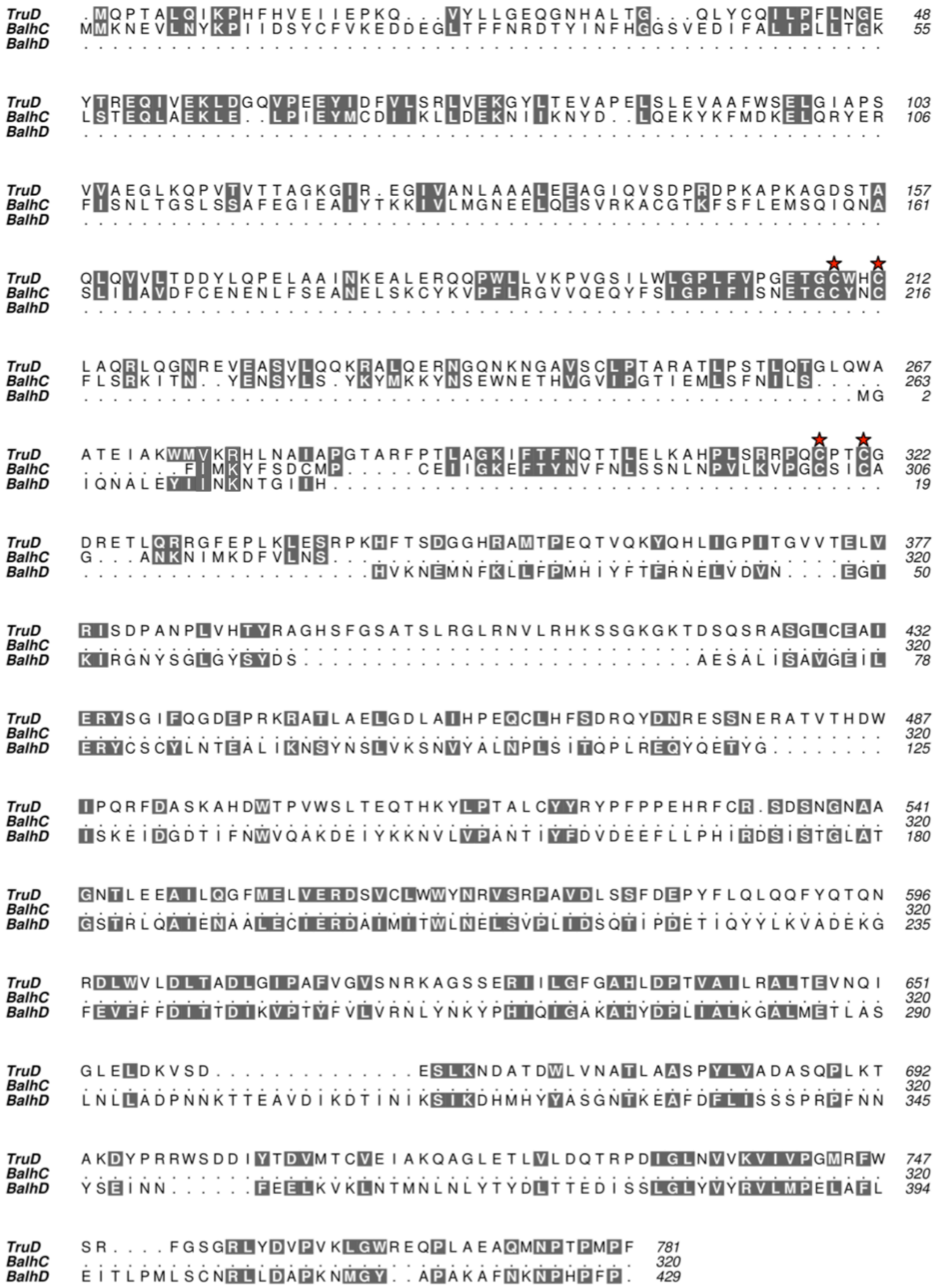

Supplementary Figure 2B: Sequence alignment of TruD with BalhC and BalhD. Cysteines coordinating the structural Zn^{2+} in TruD are marked with a red star.

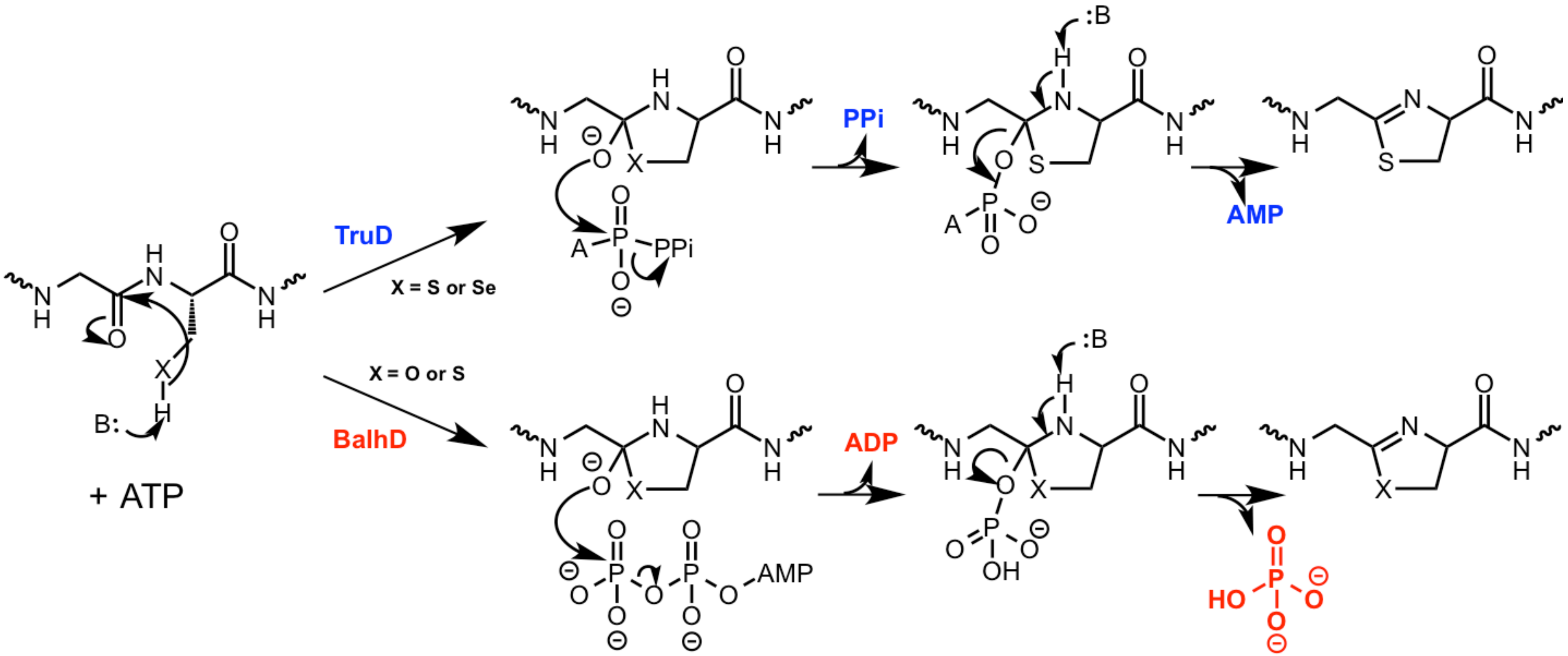

Supplementary Figure 3: Proposed mechanisms for heterocyclization reactions carried out by TruD and BalhD. Both reactions proceed via a hemiorthoamide but crucially it has been reported that BalhD phosphorylates this intermediate while our data show that TruD performs an adenylation reaction.

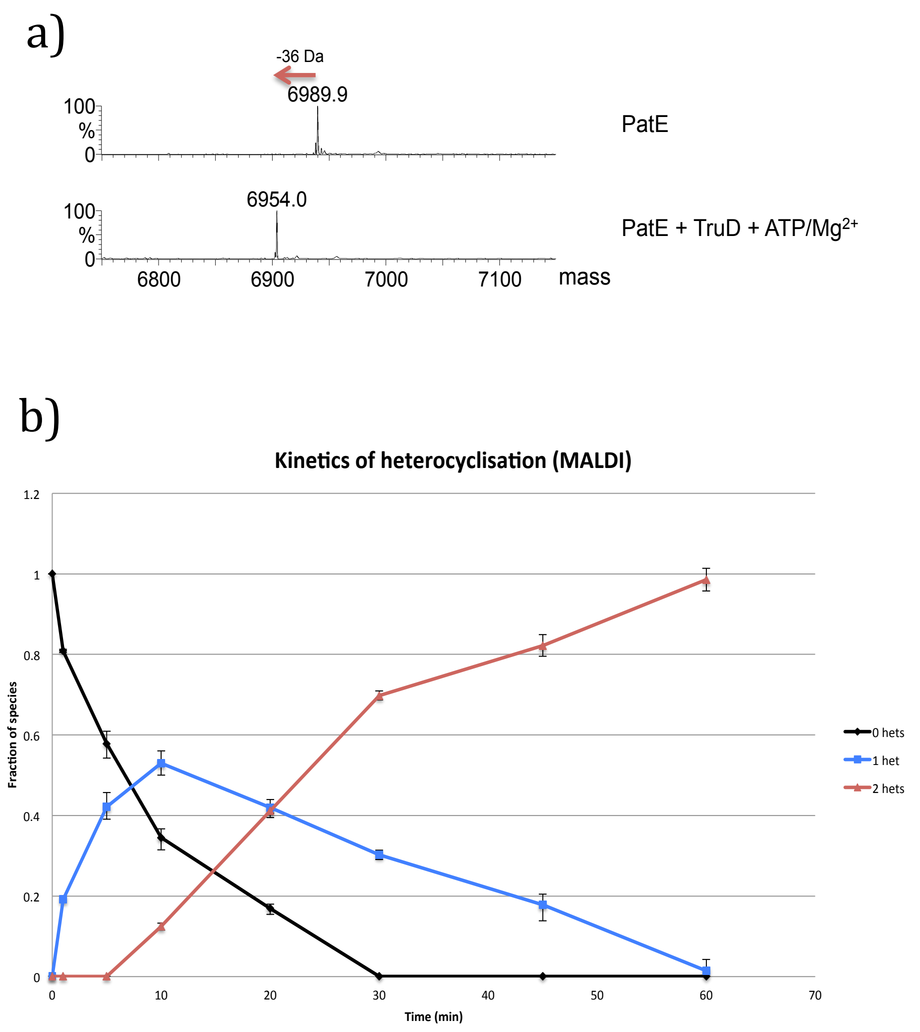

Supplementary Figure 4: a) LC-MS analysis of PatE2 when incubated at 37 °C for 16 hours with and without TruD, ATP and $MgCl_2$. Incubation with TruD results in the loss of 36 Da consistent with two water molecules. b) Time course MALDI-MS analysis of PatE2 heterocyclization following the ratios of 0, 1 and 2 heterocycles. The reaction goes to completion with 60 min at 37 °C. Each data point in the graph was obtained by three independent experiments each measured in triplicate; error bars represent errors between experiments.

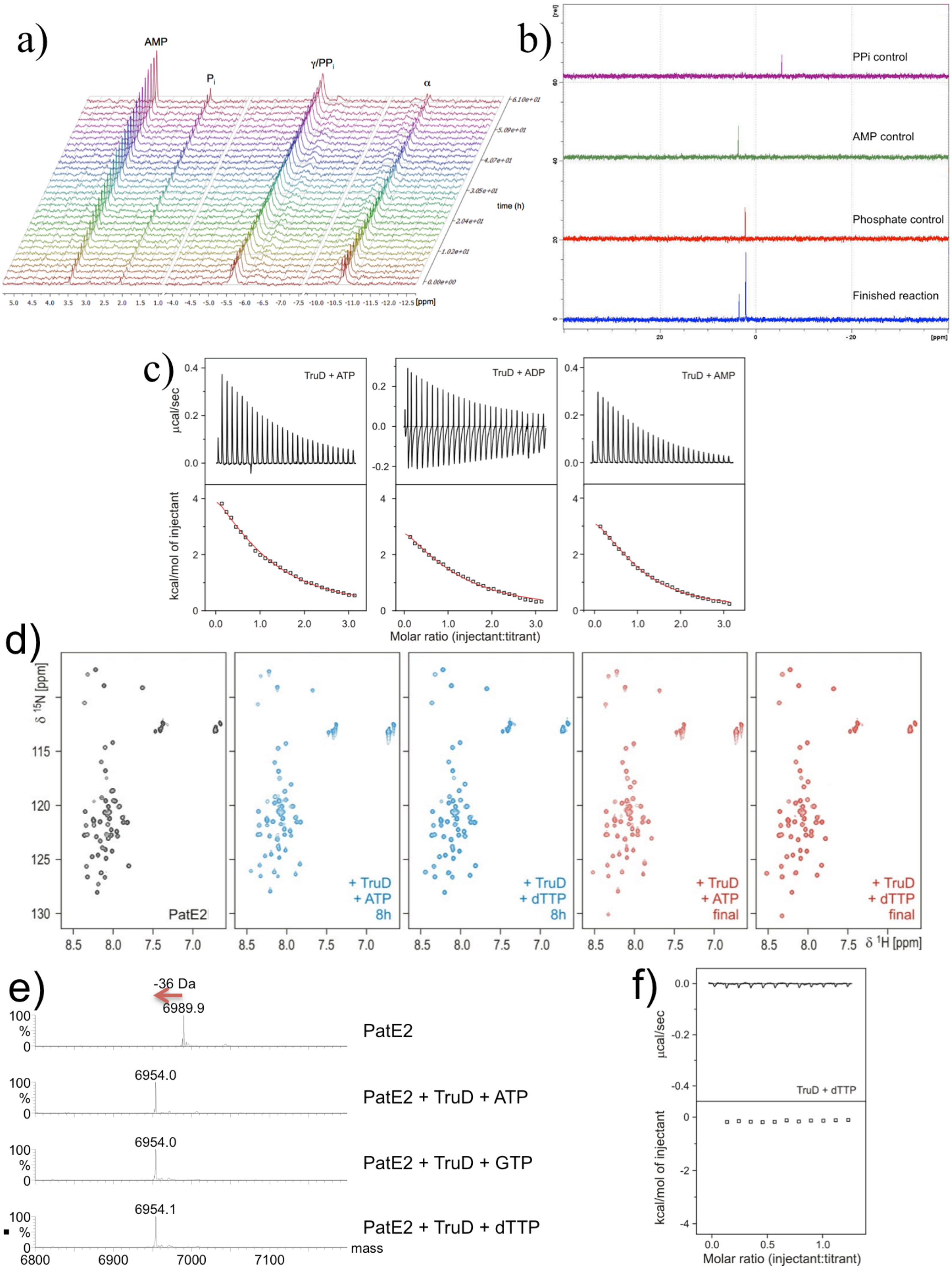

Supplementary Figure 5: a) Time course of a heterocyclization reaction by ^{31}P NMR spectra monitoring the consumption of ATP and production of AMP and PPi. The peak corresponding to AMP grows during the reaction while the peak for the α-phosphate of ATP decreases as ATP gets consumed during the reaction. A small amount of phosphate is also detectable which increases slowly over time. The signals for PPi and the γ-phosphate of ATP overlap, but this peak is the same height as that for the α-phosphate before addition of enzyme and increases while the α-phosphate

signal decreases indicating production of PPi. b) 1-D ^{31}P NMR spectrum of a heterocyclization reaction with TruD and PatE2 after incubation at 37 °C overnight (bottom). Only AMP and phosphate are observed. Control spectra for pyrophosphate, AMP and phosphate in the same buffer are shown for comparison. We interpret this as indicating that PPi has degraded under these conditions. We do not believe the mechanism has changed. c) ITC data obtained for the injection of nucleosides into TruD solutions. The top panels show raw data representing the response to injections, the bottom panels show integrated heats of injections (□) and the best fit (—) to the One-Site model (Origin). Data for ADP suggest enzyme-dependent degradation of the nucleoside. In order to obtain an apparent K_D value only the endothermic contributions to the ITC signals were integrated. d) NMR analysis of intermediate and final product obtained by TruD conversion of PatE2 in the presence of ATP and dTTP. The HSQC spectra of ^{15}N-PatE2 products are indistinguishable for both intermediate and final product generated with either nucleoside as cofactor. e) LC-MS analysis of heterocyclization reactions of PatE2 with TruD and ATP, GTP or dTTP. All three reactions show a loss of 36 Da consistent with the formation of two heterocycles. f) ITC data obtained for the injection of dTTP into a TruD solution do not report binding under these conditions.

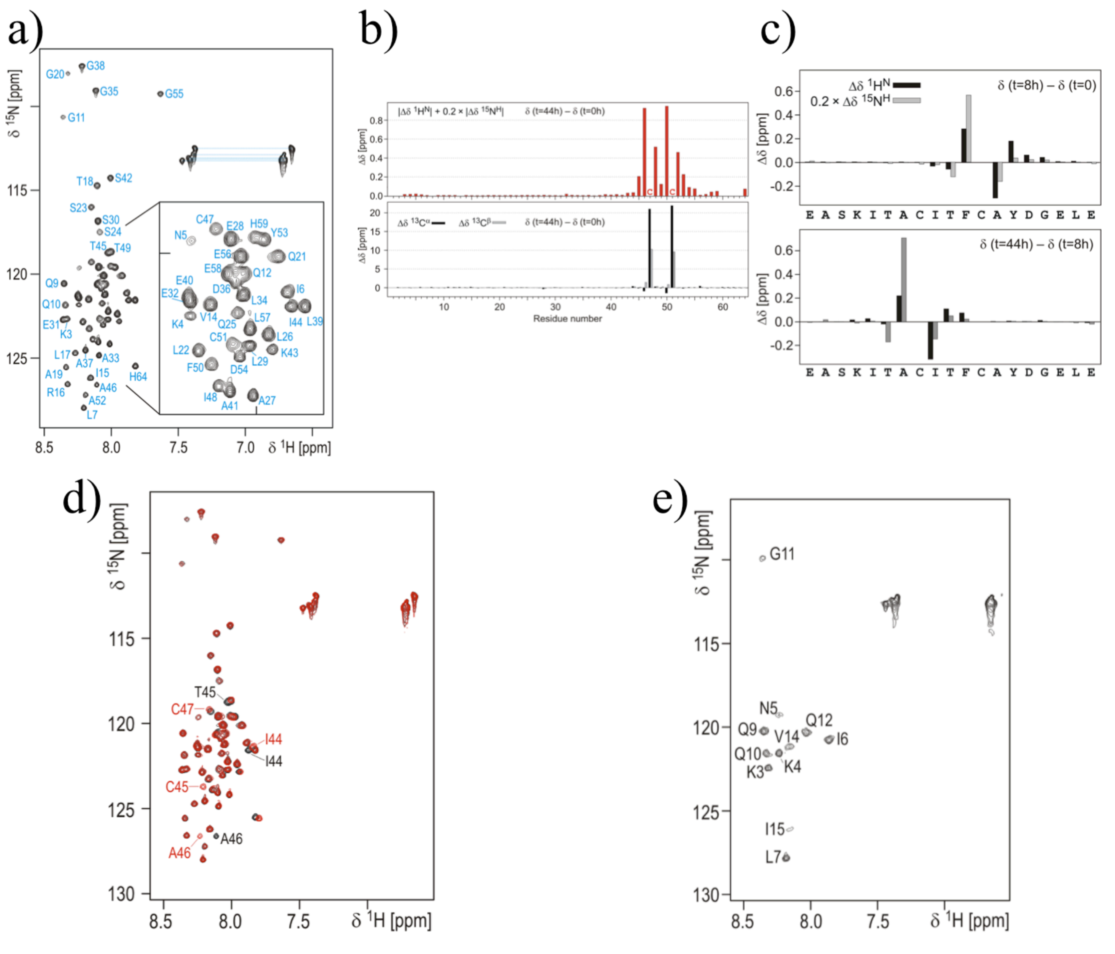

Supplementary Figure 6: a) Assigned ^{1}H, ^{15}N HSQC spectrum of PatE2, recorded at 10 °C. The sharp, poorly dispersed signals suggest that PatE exists in a natively unfolded state. b) Chemical shift changes observed for the complete conversion of PatE2 by TruD. Top, combined absolute values of chemical shift changes of amide protons and nitrogens; bottom, α and β ^{13}C chemical shift changes. ^{15}N chemical shifts are scaled by a factor of 0.2 to a magnitude comparable to ^{1}H chemical shift changes. c) Detailed amide chemical shift changes observed for the two distinct, consecutive heterocyclization steps of PatE2. d) An overlay of HSQC spectra of PatE2 (black contours) and PatE3C (red contours) highlights the very localized changes induced by the T45C mutation. C45 and A46 were assigned tentatively, and are in agreement with the observed chemical shift changes during the reaction. e) HSQC of ^{15}N-PatE2 in the presence of a 1.5 fold excess of TruD. All but the crosspeaks assigned to the N-terminal 15 residues and several Asn/Gln sidechain amide signal pairs (not labeled) are invisible due to broadening.

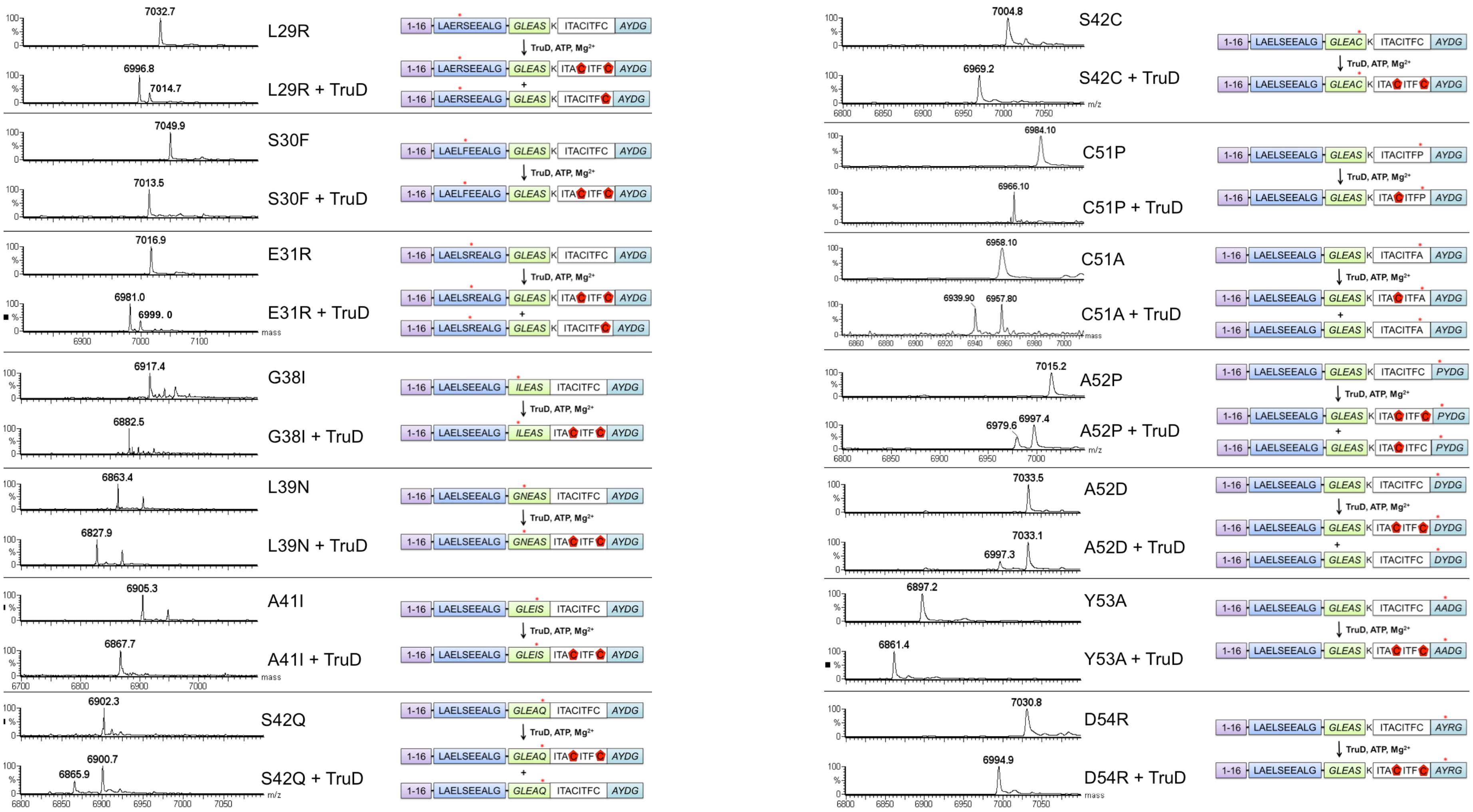

Supplementary Figure 7a: LC-MS analysis of PatE mutants after reaction with TruD. Formed heterocycles (all thiazolines) are shown as red pentagons. Their identities and positions were confirmed by MS/MS analysis.

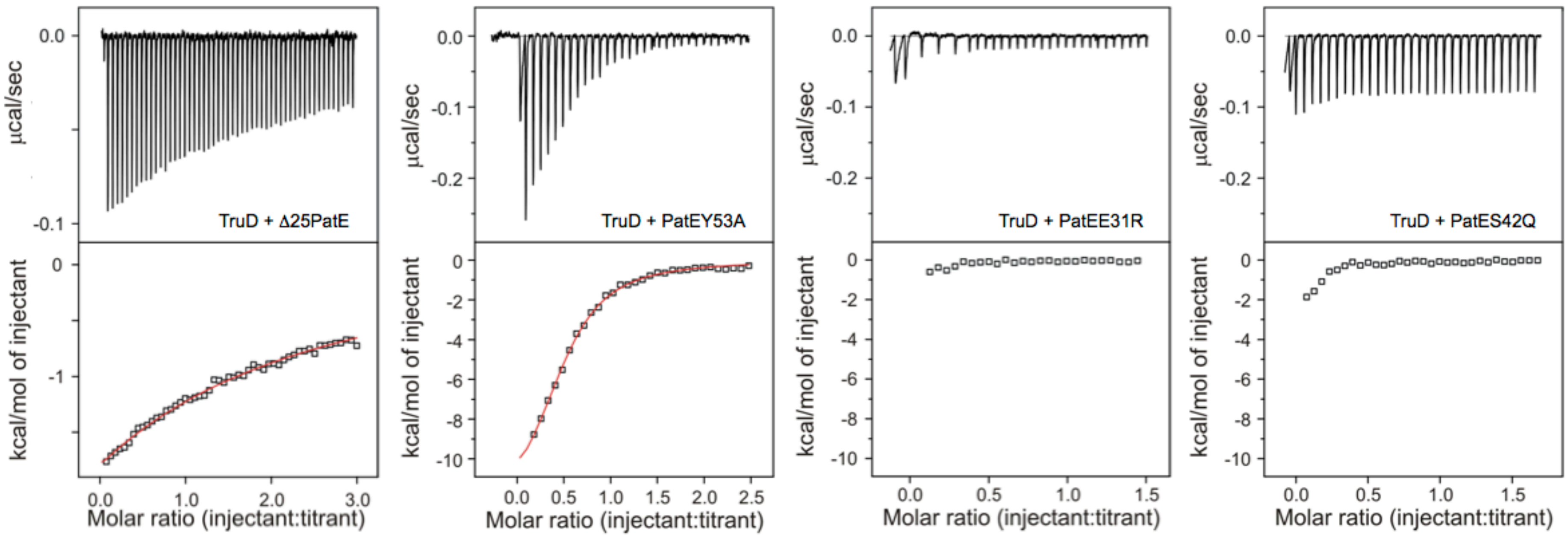

Supplementary Figure 7b: ITC data obtained for the injection of PatE variants into TruD solutions. The top panels show raw data representing the response to injections, the bottom panels show integrated heats of injections (□) and the best fit (—) to the One-Site model (Origin). Data for PatEE31R and PatES42Q could not be fitted. Obtained dissociation constants are listed in Supplementary Table 1.

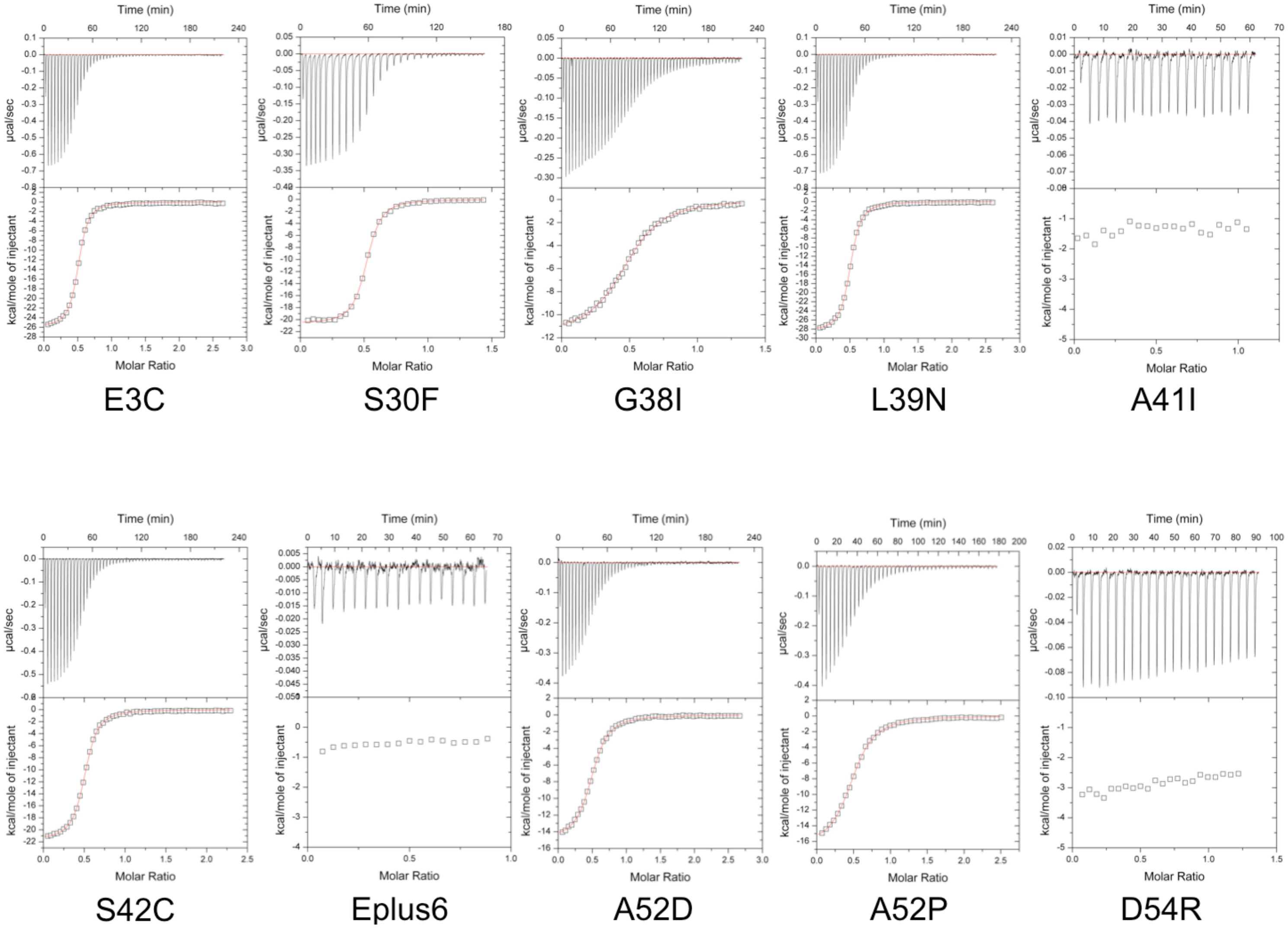

Supplementary Figure 7c: ITC data obtained for the injection of PatE variants into TruD solutions. The top panels show raw data representing the response to injections, the bottom panels show integrated heats of injections (□) and the best fit (—) to the One-Site model (Origin). Data for PatA41I, A52D and D54R could not be fitted. Obtained dissociation constants are listed in Supplementary Table 1.

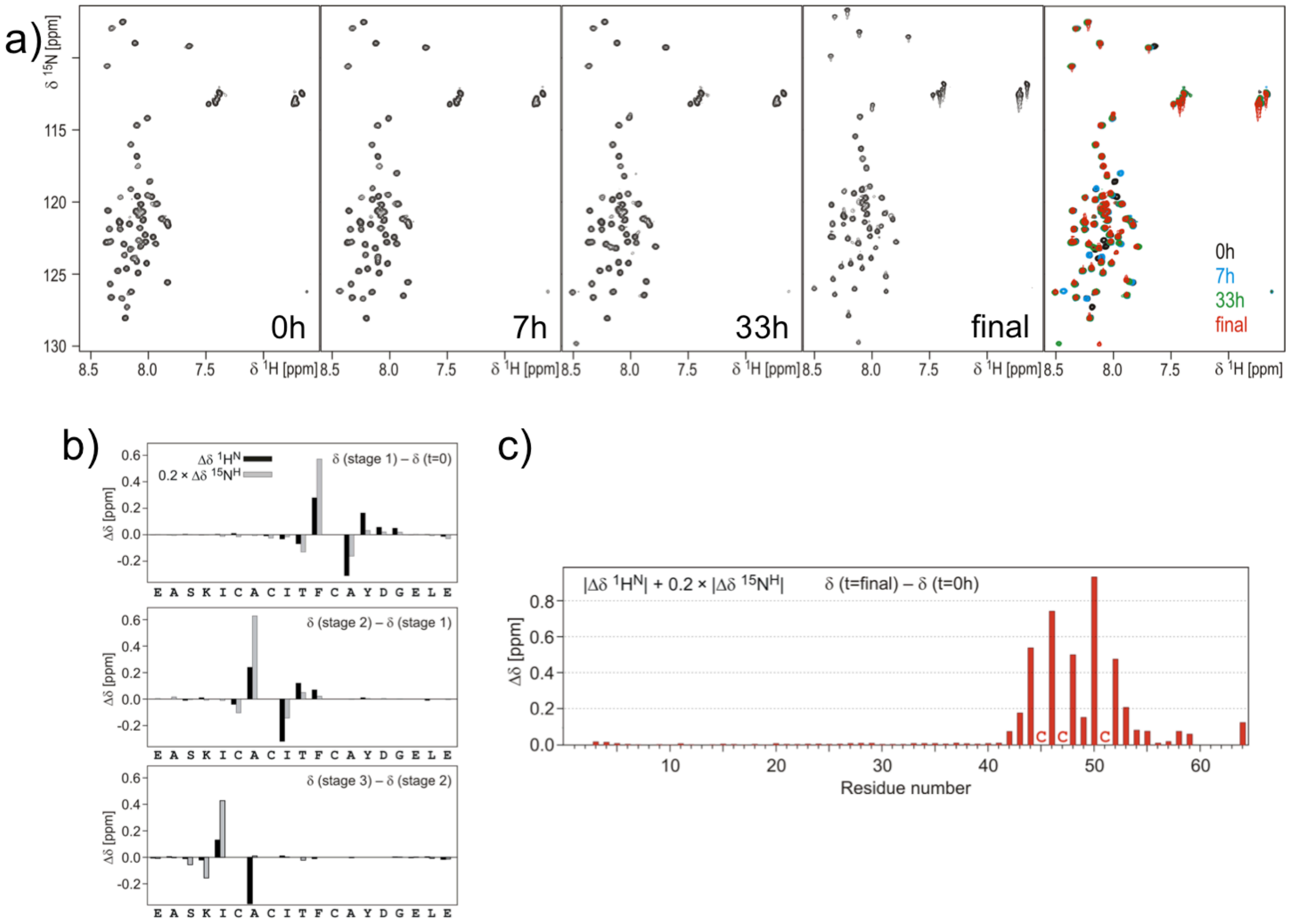

Supplementary Figure 8: a) HSQC spectra of ^{15}N-PatE3C in presence of ATP/Mg^{2+} without TruD (0 h) and after the addition of TruD and incubation at 10 °C for 7 and 33 h. The final spectrum was obtained by incubating the sample at 37 °C overnight. An overlay of all 4 spectra is shown in the right panel. b) Chemical shift changes observed for the complete conversion of PatE3C by TruD. Combined absolute values of chemical shift changes of amide protons and nitrogens; ^{15}N chemical shifts are scaled by a factor of 0.2 to a magnitude comparable to ^{1}H chemical shift changes. c) Detailed amide chemical shift changes observed for the three consecutive heterocyclization steps of PatE3C.

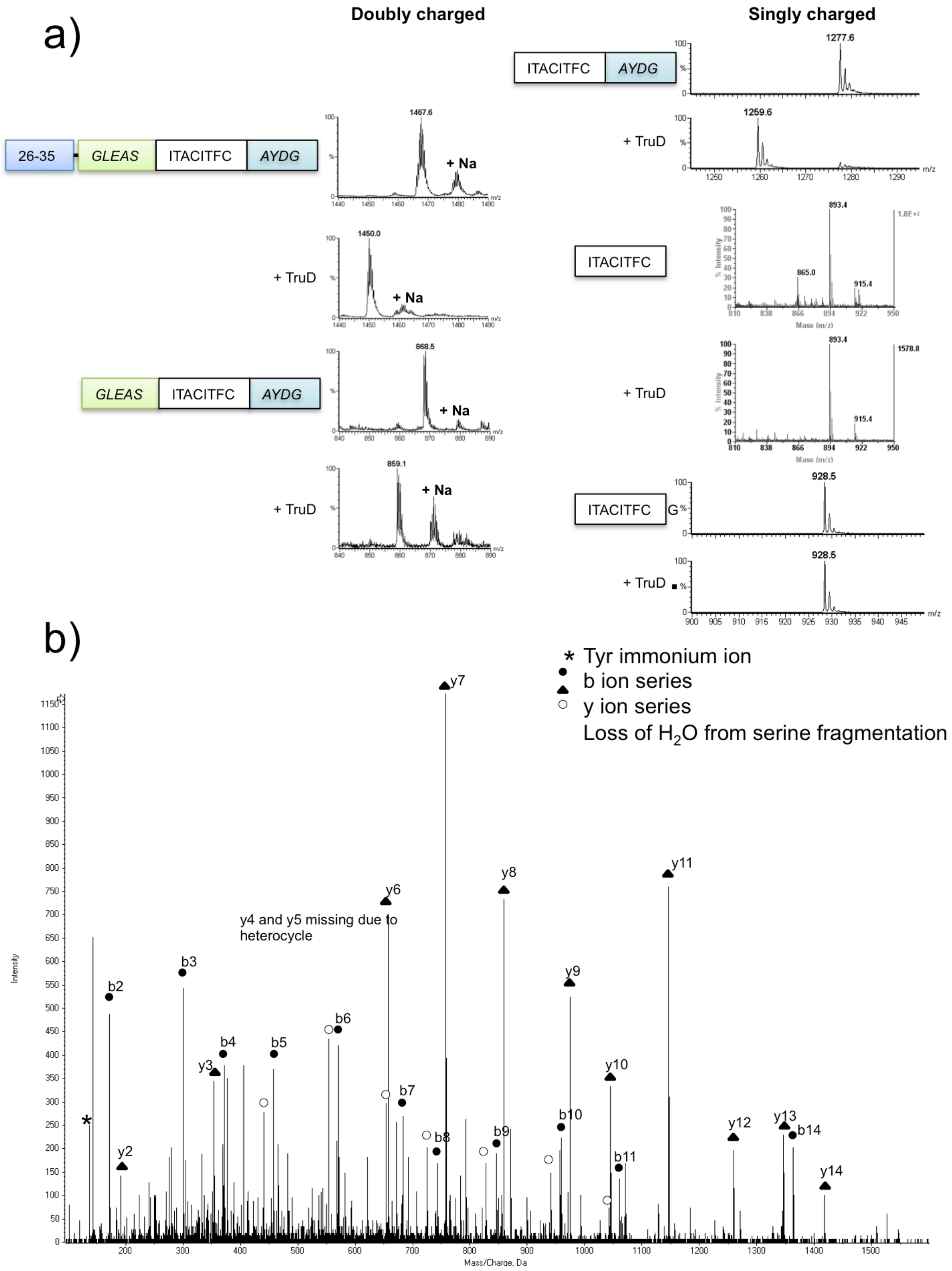

Supplementary Figure 9: a) Analysis of various PatE truncations when reacted with TruD by LC-MS. Δ25PatE gets processed on a similar timescale to wild-type protein and contains two heterocycles while Δ37 and Δ42PatE are still heterocyclized once and at a speed within an order of magnitude compared to wild-type protein. This indicated that no leader sequence was required for the enzyme to install the first heterocycle at the final core peptide position, while leader peptide residues 26-35 were essential for the formation of additional heterocycles. The core peptide alone or with an additional C-terminal glycine do not get processed by TruD. The core peptide was analyzed by MALDI-MS. b) MS/MS analysis of the heterocyclized Δ37PatE showing the position of the heterocycle is at the C-terminal cysteine. Masses for the ion series are given in supplementary Table 3.

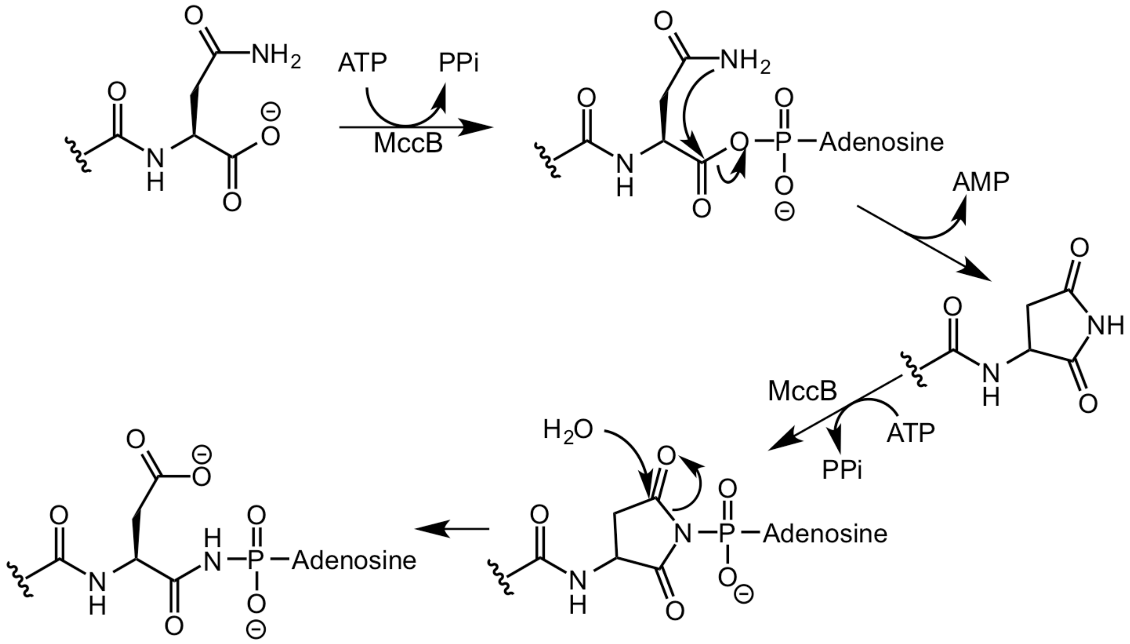

Supplementary Figure 10: Reaction mechanism for MccB. The adenylation of the C-terminus of the MccA heptapeptide (substrate, N-terminus not shown) is followed by a rearrangement, which removes AMP and results in the formation of a succinamide ring. The succinamide ring is then the substrate of a second adenylation reaction resulting in ring opening and yielding the final product.

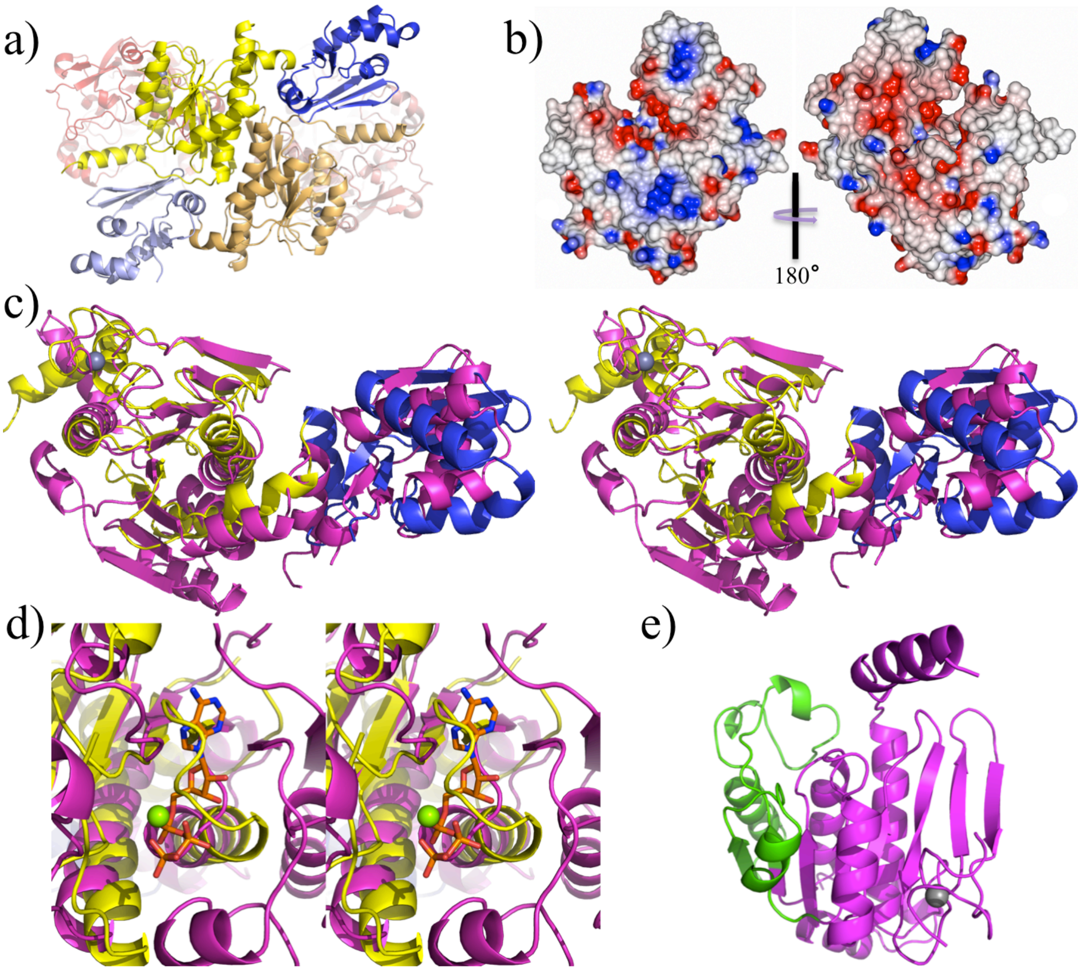

Supplementary Figure 11: a) Structure of the TruD dimer as viewed facing the extensive antiparallel dimer interface of domains 1 (blue/faded blue) and domains 2 (yellow/faded yellow). b) Electrostatic surface potential representation of TruD domain 3 showing a negatively charged cleft (front and back). c) Structural alignment of TruD (domain 1 blue, domain 2 yellow) with MccB (magenta, PDB 3H5A) shown in stereo d) Superposition of TruD domain 2 (yellow) with the ATP-bound structure of MccB (magenta, PDB 3H5N). ATP is shown as sticks, Mg^{2+} coordinated by the protein and ATP as a green sphere. e) Domain 2 of MccB. The part of the ATP binding site missing in TruD is highlighted in green.

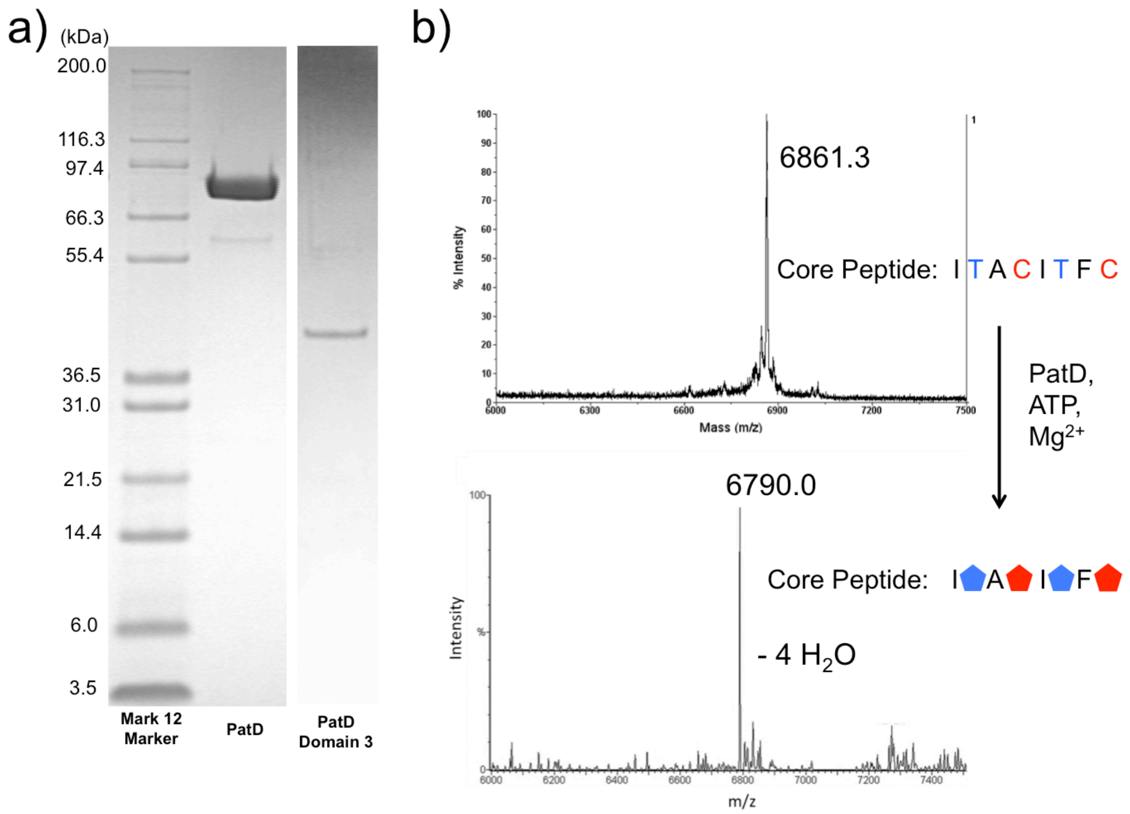

Supplementary Figure 12: a) SDS-PAGE of purified full length PatD protein and purified trypsin-digested PatD encompassing domain 3 (residues 410-784), as determined by mass fingerprinting. b) Heterocyclization of the PatE1 precursor peptide by PatD with the loss of 4 water molecules corresponding to the formation of two thiazolines (red) and two oxazolines (blue) from cysteine and threonine residues respectively. The reaction was analyzed by MALDI-MS or LC-ESI-MS.

Supplementary Table 1: PatE mutations and their effect on heterocyclization and binding

PatE		MS (No. of heterocycles)	ITC (µM)
PatE1	1-16 – 26-35 – GLEAS ITACITFC AYDG	2	0.08
PatE2	1-16 – 26-35 – GLEAS K ITACITFC AYDG	2	0.08
PatE3C	1-16 – 26-35 – GLEAS K ICACITFC AYDG	3	0.13
Δ25PatE	26-35 – GLEAS ITACITFC AYDG	2	119
Δ37PatE	GLEAS ITACITFC AYDG	1	N.D.
Δ42PatE	ITACITFC AYDG	1	N.D.
Core Peptide	ITACITFC	0	N.D.
Core peptide-G	ITACITFC G	0	N.D.
PatE+6	1-16 – 26-35 – GLEAS XXXXXX ITACITFC AYDG	2	No Binding
L29R	1-16 – 26-35 – GLEAS K ITACITFC AYDG	1 and 2	No Binding
S30F	1-16 – 26-35 – GLEAS K ITACITFC AYDG	2	0.08
E31R	1-16 – 26-35 – GLEAS K ITACITFC AYDG	1 and 2	No Binding
G38I	1-16 – 26-35 – ILEAS ITACITFC AYDG	2	0.96
L39N	1-16 – 26-35 – GNEAS ITACITFC AYDG	2	0.15
A41I	1-16 – 26-35 – GLEIS ITACITFC AYDG	2	No Binding
S42Q	1-16 – 26-35 – GLEAQ ITACITFC AYDG	0 and 2	No Binding
S42C	1-16 – 26-35 – GLEAC K ITACITFC AYDG	2	0.18
C51P	1-16 – 26-35 – GLEAS K ITACITFP AYDG	1 (Max 1)	1.5
C51A	1-16 – 26-35 – GLEAS K ITACITFA AYDG	0 and 1 (Max 1)	0.3
A52P	1-16 – 26-35 – GLEAS K ITACITFC PYDG	1 and 2	0.53
A52D	1-16 – 26-35 – GLEAS K ITACITFC DYDG	0 and 2	0.38
Y53A	1-16 – 26-35 – GLEAS K ITACITFC AADG	2	1.1
D54R	1-16 – 26-35 – GLEAS K ITACITFC AYRG	2	Very weak

Positions of point mutations are indicated by red stars, positions of heterocycles by red pentagons. Faded red pentagons indicate that the formation of the heterocycle was incomplete. N.D. - Not Determined as substrate too insoluble in buffer (150 mM NaCl, 10 mM HEPES, 5 mM $MgCl_2$, 1 mM TCEP) to reliably measure affinity.

Supplementary Table 2: Data collection and refinement statistics (SAD)
*1 crystal user per structure *Values in parenthesis are for highest-resolution shell.

	SAD data set	Dataset used for refinement
**Data collection**		
Space group	$I4_122$	$I4_122$
Cell dimensions		
a, *b*, *c* (Å)	137.88, 137.88, 279.84	138.27, 138.27, 280.39
α , *β*, γ (°)	90, 90, 90	90, 90, 90
Wavelength (Å)	1.28	1.28
Resolution (Å)	123.68 – 3.33 (3.41 – 3.33)	97.77 – 2.90 (3.06 – 2.90)
R_{sym} or R_{merge}	13.6 (59.3)	12.8 (56.6)
I / s*I*	17.2 (6.9)	6.4 (1.9)
Completeness (%)	99.8 (99.6)	93.0 (95.1)
Redundancy	25.1 (27.1)	5.3 (5.2)
**Refinement**		
Resolution (Å)		77.43 – 2.90
No. reflections		26,506
R_{work} / R_{free}		0.185 / 0.237
No. atoms		5,472
Protein		5,470
Ligand/ion		1
Water		1
B-factors		81.19
Protein		81.19
Ligand/ion		54.37
Water		58.49
R.m.s. deviations		
Bond lengths (Å)		0.009
Bond angles (°)		1.343

Supplementary Table 3: Masses of ions highlighted in Supplementary Figure 9b.

	Int cleav (-GLE)	b ion	y ion	Imm (Tyr)	Ser frag (- 18)
G					
L		171.1075			
E		300.1521			
A		371.1910	1417.6279		
S		458.2221	1346.5984		440.2113
I		571.3055	1259.5564		553.2961
T		672.3539	1146.4718		654.3467
A		743.3969	1045.4237		725.3830
C		846.4079	974.3822		828.3953
I		959.4938	871.3690		941.4811
T		1060.5434	758.2866		1042.5370
F			657.2332		
*hetC					
A		1363.6663			
Y			354.1267	136.0697	
D			191.0622		
G					

Methods

Protein cloning, expression and purification

Codon optimized full-length TruD with an N-terminal TEV protease-cleavable His_6-tag was purchased from DNA2.0 in the proprietary pJexpress411 vector. It was expressed in *Escherichia coli* BL21 (DE3) grown in autoinduction medium [1] for 48 h at 20 °C. Cells were harvested by centrifugation at 4,000 x g, 20 °C for 15 min and resuspended in lysis buffer (500 mM NaCl, 20 mM Tris pH 8.0, 20 mM Imidazole and 3 mM β-mercaptoethanol (BME)) with the addition of complete EDTA-free protease inhibitor tablets (Roche) and 0.4 mg DNase g^{-1} wet cells (Sigma). Cells were lysed by passage through a cell disruptor at 30 kPSI (Constant Systems Ltd) and the lysate was cleared by centrifugation at 40,000 x g, 4 °C for 20 min. Cleared lysate was applied to a Ni-NTA (Qiagen) column equilibrated with lysis buffer and protein eluted with lysis buffer containing 250 mM imidazole. The protein was then passed over a desalting column (Desalt 16/10, GE Healthcare) in 100 mM NaCl, 20 mM Tris pH 8.0, 20 mM imidazole, 3 mM BME. Tobacco etch virus (TEV) protease was added to the protein at a mass-to-mass ratio of 1:10 and the protein digested for 2 h at 20 °C to remove the His_6-tag. Digested protein was passed over a second Ni-column and the flow-through loaded onto a monoQ 10/100 GL column (GE Healthcare) equilibrated in 100 mM NaCl, 20 mM Tris pH 8.0, 3 mM BME. Protein was eluted from the monoQ column through a linear NaCl gradient, eluting at 250 mM NaCl. Finally, the protein was subjected to size-exclusion chromatography (Superdex™ 200, GE Healthcare) in 150 mM NaCl, 10 mM HEPES pH 7.4, 1 mM TCEP, and concentrated to 8 mg mL^{-1}. All TruD point mutants were produced using the Phusion® site-directed mutagenesis kit (Finnzymes) following the manufacturer's protocol. All mutant proteins were expressed and purified as above.

Our engineered PatE precursor peptide containing a single core peptide was produced by heterologous expression from the pBMS vector (a gift from H. Liu) with a C-terminal His_6-tag and expressed in *Escherichia coli* BL21 (DE3) cells grown in auto induction medium [1] for 24 h at 30 °C where the protein was driven to inclusion bodies. Cells were harvested by centrifugation at 4,000 x g for 15 min at 20 °C. Cells were re-suspended in urea lysis buffer (8 M urea, 150 mM NaCl, 20 mM Tris pH 8.0, 20 mM Imidazole and 3 mM BME and lysed by sonication at 15 microns for 4 min (SoniPrep 150, MSE). The lysate was cleared by centrifugation at 40,000 x g, 20 °C

for 20 min followed by passage through a 0.45 μm filter. The cleared lysate was applied to a Ni-sepharose FF column (GE Healthcare) equilibrated with urea lysis buffer and protein eluted with urea lysis buffer containing 250 mM imidazole. The eluate was then supplemented with 10 mM DTT and incubated at room temperature for 30 min before size-exclusion chromatography (Superdex 75, GE Healthcare) in 150 mM NaCl, 10 mM HEPES pH 7.4, 1 mM TCEP. Peak fractions were pooled and concentrated to 1 mM.

PatE for NMR studies was produced as above, except that *E. coli* were grown in M9 minimal medium [2] prepared with $^{15}NH_4Cl$ (Sigma) and additionally with $^{13}C_6$-glucose (Sigma) for doubly labeled protein. Cells were grown to an optical density at 600 nm of 0.6, at which point expression was induced by addition of 0.5 mM IPTG and grown over night at 30 °C.

Heterocyclization reactions

For all heterocyclization reactions for MS analysis, 100 μM substrate (PatE, PatE mutants and peptides) was incubated with 0.5 μM enzyme in heterocyclization buffer (150 mM NaCl, 10 mM HEPES pH 7.4, 1 mM TCEP, 5 mM ATP, 5 mM $MgCl_2$) for 60 min, 70 min or 16 h at 37 °C. Samples were analyzed by ESI or MALDI MS (LCT, Micromass or 4800 MALDI TOF/TOF Analyzer, ABSciex). With the exception of PatE, and PatE mutants all peptides were purchased from Peptide Protein Research Ltd.

For heterocyclization reactions to be monitored by NMR the PatE concentration was increased to 200 μM and 5 μM TruD was used and the incubation temperature reduced to 10 °C. For reaction endpoints analyzed by ^{31}P NMR samples were incubated at 20 °C for 8 h, with the exception of the sample shown in Figure S11, which was incubated at 37 °C for 16 h.

AMP production assay

All chemicals and enzymes were purchased from Sigma-Aldrich except PatE and TruD, which were self-prepared.

The assay is based on coupling the TruD-dependent formation of AMP to the lactate dehydrogenase oxidation decay of NADH with NADH concentration monitored by 340 nm absorbance. The enzyme assays were performed in TruD sample buffer (150

mM NaCl, 10 mM HEPES pH 7.4, 1 mM TCEP) in a total volume of 400 μL at 20 °C containing 0.25 mM NADH, 5 mM $MgCl_2$, 0.6 mM ATP sodium salt, 0.6 mM phosphoenolpyruvate, 10 U (1.5 μL) of lactate dehydrogenase and pyruvate kinase mixture (900-1400 and 600-1000 units mL^{-1}, respectively) and 46 μM substrate PatE, each added to the assay in this order. The assays were initialized by adding 10 μM TruD in the absence or presence of 5 U myokinase to respectively monitor the background decay of NADH at 340 nm or the decay of NADH coupled with the production of AMP catalyzed by TruD. The decay of NADH concentration were recorded for 40 min and calculated by using extinction coefficient for NADH (ε340 = 6220 $M^{-1}cm^{-1}$).

MS analysis

LC-MS was performed on a Waters LC-MS system (LCT mass spectrometer and 2795 HPLC) using a Waters MassPrep column (2.1 x 10 mm). Solvent B was 0.1% formic acid and Solvent A was MeCN containing 0.1% formic acid. Gradient 0 - 0.5min 98 % B, 0.5 - 2.5 min linear to 2 % B, 2.5 - 4.5 min 2 % B, 4.5 - 4.6 min linear to 98 % B, 1.6 - 12 min 98 % B at 0.05 ml min^{-1}. The following conditions were used on the mass spectrometer: ESI +ve, capillary voltage 3.5 kV, cone voltage 40 V, mass range 500-2500 m/z, RF lens 500. The spectra were combined across the eluted protein peak and the charged ion series processed using Water's MaxEnt algorithm to give protein mass, using peak width at half height on the strongest peak in the ion envelope. The data was calibrated externally against horse heart myoglobin (16,951.5 Da).

MALDI MS was acquired using a 4800 MALDI TOF/TOF Analyzer (ABSciex, Foster City, CA) equipped with a Nd:YAG 355 nm laser in linear mode and calibrated using the $[M+H]^+$ and $[M+2H]^{2+}$ peaks of ubiquitin. The spot was analyzed in positive MS mode between 3000 and 10000 m/z, by averaging 1000 laser spots.

Fragmentation MS of the peptides were carried out on an ABSciex 5600 mass spectrometer with Eksigent nanoLC and Eksigent nanoflex cHiPLC column system using C18 column chemistry. Trap and elute methodology was used with a 6 minute trap wash. Trapping solvent A was 98 % water, 2 % MeCN, 0.05 % Trifluoroacetic acid. Gradient solvent A was 98 % water, 2 % MeCN, and 0.1 % formic acid and gradient solvent B was 98 % MeCN, 2 % water, and 0.1 % formic acid. Gradient 0 -

0.5 min 5 % B, 0.5 - 5 min linear to 40 % B, 5 - 6 min linear to 95 % B, 6 - 9 min 95 % B, 9 - 10 min linear to 2 % B, hold at 2 % B until 20 mins at 400 nl min^{-1}, and 45 °C. The following conditions were used on the mass spec: ESI +ve, 0.25 s MS accumulation and 0.15 s MSMS accumulation, collision energy of 45 V with rolling collision energy optimization applied. The data was calibrated externally prior to analysis with 8 peptides from a tryptic digest of 25 fmol μl^{-1} Beta-galactosidase.

MALDI mass spectrometry for the time course was carried out on an ABSciex 4800 MALDI TOF/TOF mass spectrometer. 0.5 µl of sample was co-spotted with 0.5 µl of matrix (10 mg ml^{-1} alpha cyano- 4-hydroxycinnamic acid in 50% MeCN and 50% 0.1 % TFA) and the sample left to dry. Spectra were collected over the range 500 - 4000m/z with 20 subspectra of 50 shots accumulated randomly across the spot. The data was calibrated externally prior to analysis with 6 peptides from ABSciex standard 6 peptide mix (PN 4465940).

ITC and data analysis

ITC experiments were carried out using a VP-ITC instrument (MicroCal) in TruD sample buffer (150 mM NaCl, 10 mM HEPES pH 7.4, 1 mM TCEP) at 20 or 25 °C. Protein concentrations were determined using absorbance at 280 nm and predicted extinction coefficients (ProtParam, ExPASy) used. The concentration of PatEY53A, which lacks absorbance at 280 nm, was based on absorbance at 230 nm. All solutions were dialyzed overnight. Nucleotides and the Δ25 peptide were dissolved in TruD sample buffer and the pH was adjusted with small amounts of 1 M NaOH to match the buffer. Cell and syringe solutions were degassed at 18 or 23 ºC for 15 min. Titrations were performed as follows: one injection of 2 µl followed by injections of 5-10 µl at an injection speed of 0.5 µl/min. The stirring speed was 307 rpm; the delay between the injections was 4-6 min. To take into account heats of dilution, blank titrations were performed by injecting ligand solution into TruD sample buffer and the averaged heat of dilution was subtracted from the main experiment. Blank titrations were omitted in cases of saturated binding where the averaged residual signal of the last injections was used to determine the heat of dilution. Raw data were processed using MicroCal Origin software. The baseline was adjusted and integrations were carried out manually. Data were non-linearly fitted to the One-site model (Origin). We repeatedly observed a stoichiometry in ITC of ~ 0.48 indicating a systematic error

in peptide concentration or half the enzyme is inactive or one substrate binds per TruD dimer. K_D values are insensitive to cell (TruD) concentrations, and changes only slightly if syringe (peptides) concentrations are adjusted. Thus we believe the K_D values are sound but are cautious to avoid over interpreting stoichiometry.

NMR

NMR experiments were performed at 10 °C on a Bruker DRX500 spectrometer equipped with a 5mm TXIz probe. The instrument was controlled by Topspin (Bruker). The sample for the binding experiment contained 100 μM *u*-^{15}N-PatE precursor peptide in heterocyclization buffer supplemented with 5 % D_2O. Aliquots of 646 μM TruD were added to final concentrations of 50, 100 and 150 μM, and HSQCs were recorded after each addition. For heterocyclization monitoring, the samples contained 200 μM *u*-^{15}N-PatE precursor peptide and were incubated at 10 °C with 5 μM TruD in heterocyclization buffer with 5% D_2O, and spectra were recorded at progressively longer intervals (initially 0.5 h, then 1 h, then 2 h). ^{1}H, ^{15}N HSQC spectra were acquired with Watergate water suppression [3] at 1024×128 points and a digital resolution of 9.8 Hz and 19.0 Hz for the ^{1}H and ^{15}N dimension, respectively. For sequential assignment, a 1.0 mM sample of *u*-^{13}C, ^{15}N-PatE precursor peptide in heterocyclization buffer was prepared. HNCACB and CBCA(CO)NH spectra with Watergate water suppression were acquired with 1024×60×128 points for the ^{1}H, ^{15}N and ^{13}C dimension, respectively. Equivalent spectra were recorded for fully heterocyclized *u*-^{13}C, ^{15}N-PatE (concentration 1.0 mM) and a sample where the enzymatic transformation was interrupted after 8 h by gel filtration on a Superdex S75 column (GE Healthcare) in TruD sample buffer (see ITC experimental section), thus allowing assignment of the reaction intermediate. All spectra were processed with NMRPipe [4] and analyzed with CCPN Analysis 2.1 [5].

^{31}P NMR experiments were performed at 10 °C on a Bruker AVANCE III spectrometer equipped with a 5mm BBFO+ (Smart) probe and required the substitution of sample buffer TCEP with DTT. The instrument was controlled by and spectra were processed with Bruker Topspin. ^{31}P NMR spectra were recorded with broadband power-gated 1H decoupling and relaxation delay D1 = 1.5s.

Crystallization, data collection, and crystallographic analysis

Crystals of TruD were obtained in 1.08 M sodium malonate, 1.92 % (v/v) Jeffamine ED2001, 0.1 M HEPES pH 7.5. The crystals were cryoprotected in 30 % sucrose and flash-cooled in liquid nitrogen. These crystals belonged to space group $I4_122$ with cell dimensions a = b = 138.3 Å, c = 280.4 Å. Diffraction data was collected at Diamond beamline IO3 at 100 K and processed with xia2 [6]. Phases were determined with Phenix using the low-resolution, high-redundancy dataset collected at the Zn-edge [7]. An initial model was built with Buccaneer implemented in the CCP4 program suite [8]. This initial model was used as a search model for molecular replacement with PHASER [9] using the high-resolution dataset. Complete manual rebuilding was performed with COOT [10] and refinement was performed using REFMAC5 [11] implemented in the CCP4 program suite [8]. The statistics of data collection and refinement are summarized in Supplementary Table 1. All molecular graphics figures were generated with the program Pymol (DeLano Scientific, LLC). The final model contains residues 3-144, 156-237, 250-343, 355-378, 423-656 and 667-781; missing residues were presumed disordered. Crystal symmetry creates an antiparallel dimer (Figure 4a) in which both domains 1 and 2 make extensive inter-subunit contacts burying 2946 Å2 (Figure S11), which the structure interface analysis webserver PISA [11] predicts is stable, consistent with our gel filtration data.

Domain 1 has a box like shape, on one face a three-stranded β-sheet and on the opposite face two α-helices, a small two stranded β-sheet and an α-helix each form another face. Domain 2 contains a central six-stranded mixed parallel and antiparallel β-sheet flanked on both sides by two α-helices. This domain contains a tetrahedral zinc ion coordinated by four sulfur atoms from two CXXC motifs and the site is close to the domain 2 domain 3 boundary. Domain 3 also has a central β-sheet flanked by helices, but here the sheet is a five-stranded antiparallel sheet. One face of the central sheet is flanked by four α-helices and loops which together contact domain 2. The other face of the sheet contains a helical bundle comprising of eight α-helices and a two-stranded β-sheet.

References

[1] F. W. Studier, *Protein Expr Purif* **2005**, *41*, 207-234.
[2] F. C. Neidhardt, P. L. Bloch, D. F. Smith, *J Bacteriol* **1974**, *119*, 736-747.
[3] M. Piotto, V. Saudek, V. Sklenar, *J Biomol NMR* **1992**, *2*, 661-665.
[4] F. Delaglio, S. Grzesiek, G. W. Vuister, G. Zhu, J. Pfeifer, A. Bax, *J Biomol NMR* **1995**, *6*, 277-293.

[5] W. F. Vranken, W. Boucher, T. J. Stevens, R. H. Fogh, A. Pajon, M. Llinas, E. L. Ulrich, J. L. Markley, J. Ionides, E. D. Laue, *Proteins* **2005**, *59*, 687-696.
[6] G. Winter, *J Appl Cryst*, *43*, 186-190.
[7] P. D. Adams, P. V. Afonine, G. Bunkoczi, V. B. Chen, I. W. Davis, N. Echols, J. J. Headd, L. W. Hung, G. J. Kapral, R. W. Grosse-Kunstleve, A. J. McCoy, N. W. Moriarty, R. Oeffner, R. J. Read, D. C. Richardson, J. S. Richardson, T. C. Terwilliger, P. H. Zwart, *Acta Crystallogr D Biol Crystallogr* **2010**, *66*, 213-221.
[8] M. D. Winn, C. C. Ballard, K. D. Cowtan, E. J. Dodson, P. Emsley, P. R. Evans, R. M. Keegan, E. B. Krissinel, A. G. Leslie, A. McCoy, S. J. McNicholas, G. N. Murshudov, N. S. Pannu, E. A. Potterton, H. R. Powell, R. J. Read, A. Vagin, K. S. Wilson, *Acta Crystallogr D Biol Crystallogr* **2011**, *67*, 235-242.
[9] a) A. J. McCoy, R. W. Grosse-Kunstleve, L. C. Storoni, R. J. Read, *Acta Crystallogr D Biol Crystallogr* **2005**, *61*, 458-464; b) L. C. Storoni, A. J. McCoy, R. J. Read, *Acta Crystallogr D Biol Crystallogr* **2004**, *60*, 432-438.
[10] P. Emsley, B. Lohkamp, W. G. Scott, K. Cowtan, *Acta Crystallogr D Biol Crystallogr* **2010**, *66*, 486-501.
[11] G. N. Murshudov, A. A. Vagin, E. J. Dodson, *Acta Crystallogr D Biol Crystallogr* **1997**, *53*, 240-255.
